# Structural Origin of Morphotropic Phase Boundary in Advanced Perovskite Ferroelectric Oxides

**DOI:** 10.1021/jacs.5c22401

**Published:** 2026-02-13

**Authors:** Yajun Yue, Fengjin Qu, Giuseppe Viola, Bing Han, Marcin Krynski, Takashi Honda, Qifeng Zheng, Zimeng Hu, Isaac Abrahams, Haixue Yan

**Affiliations:** † School of Chemistry, 12451 South China Normal University, Guangzhou, Guangdong 510006, China; ‡ Spallation Neutron Source Science Center, 561810 China Spallation Neutron Source, Dongguan, Guangdong 523803, China; § School of Engineering and Materials Science, 4617 Queen Mary University of London, Mile End Road, London E1 4NS, U.K.; ∥ Key Laboratory of Inorganic Functional Materials and Devices, Shanghai Institute of Ceramics, Chinese Academy of Sciences, 588 Heshuo Road, Jiading, Shanghai 201899, People’s Republic of China; ⊥ Faculty of Physics, 49566 Warsaw University of Technology, ul. Koszykowa 75, Warsaw 00-662, Poland; # J-PARC Center, High Energy Accelerator Research Organization (KEK), Tokai, Ibaraki 319-1106, Japan; ∇ Department of Chemistry, 4617 Queen Mary University of London, Mile End Road, London E1 4NS, U.K.; ○ Institute of High Energy Physics, Chinese Academy of Sciences, Beijing 100049, China

## Abstract

Ferroelectric oxides PbZr_1–*x*_Ti_*x*_O_3_ (PZT) with the ABO_3_ perovskite structure exhibit exceptional polarization responses near their morphotropic phase boundary (MPB), yet the chemical origin of this behavior remains unclear. Here, we show that, in a prototypical composition, 0.05Pb­(Mn_1/3_Sb_2/3_)­O_3_–0.95PbZr_0.52_Ti_0.48_O_3_, this origin arises from coupled effects of B-site chemical ordering and multi-ion displacement heterogeneity-related disordering. Pronounced anti-self-clustering of Zr and Ti forms a short-range chemical ordering driven by the mismatch between ionic Zr–O and more covalent Ti–O bonds, generating a soft–hard compatible BO_6_ network that reduces local stress, which facilitates polarization rotation and switching. Simultaneously, A-site, B-site, and oxygen ions display significant, directionally distinct off-center displacements, producing continuous local monoclinic polar states (M_A_–M_B_) with coplanar polarization vectors and nanoscale domains with mobile walls. These results show that PZT’s extraordinary response emerges from a unity-of-opposites relationship that balances rigidity and flexibility through compatible bonding and multi-ion displacements, offering guidance for designing high-performance ferroelectrics.

## Graphical abstract

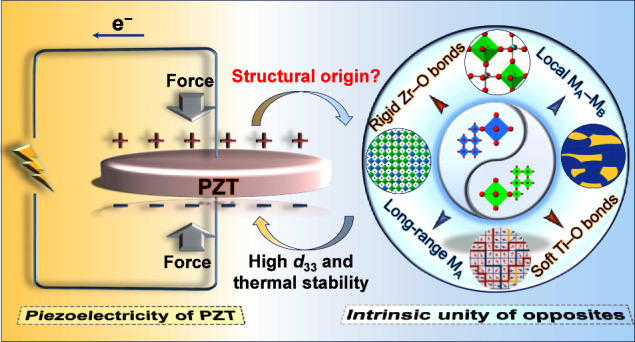

## 1 Introduction

Piezoelectric ceramics, capable of interconverting mechanical and electrical energy, are widely used in modern electromechanical technologies, including actuators, sensors, sonar systems and ultrasound imaging devices.^1,2^ Perovskite structured PbZr_1–*x*_Ti_*x*_O_3_ (PZT) oxides with compositions near the morphotropic phase boundary (MPB, *x* ≈ 0.45 – 0.48) show outstanding electromechanical coupling ability, high piezoelectric coefficients (e.g., *d*_33_ ∼ 200 – 600 pC/N), and excellent thermal stability, which far exceed other known piezoelectric materials.^3,4^ Although lead-containing compositions raise environmental concerns, at present, PZT remains the most important piezoelectric ceramic in both scientific research and industrial applications.^5–7^ Understanding the chemical origin of the exceptional piezoelectric performance in MPB compositions is crucial not only for guiding the development of novel piezoceramics including perovskite lead-free alternatives, but also for offering insights into relaxor-like ferroelectrics, which show higher piezoelectric coefficients than PZT with lower thermal stability.^8–12^

The piezoelectric performance of a poled ceramic can be described by the piezoelectric coefficient *d*_33_, expressed as^13^

$$d_{33}=2P_{r}Q\varepsilon_{33}$$ 1

where *P*_r_ is the remnant polarization, reflecting both the strength and stability of the overall polarization retained in a piezoelectric after poling; *Q* represents the electrostriction coefficient, reflecting a material’s ability to deform in response to an electric field; and ε_33_ is the permittivity, which is strongly correlated with the change of polarization under an applied electric field. These three parameters are intricately linked to both long- and short-range polar structures, as well as their evolution under applied electric fields. It is worth noting that the long-range structure refers to the crystallographically ordered average structure, while the short-range structure captures atomic arrangements at the nanometer scale. A comprehensive understanding of piezoelectricity requires simultaneous considerations of structural features in both ranges and their coupling during field-induced changes.

In the long-range regime, the prevailing MPB theory considers the coexistence of multi long-range ferroelectric phases, namely tetragonal (T, space group *P*4*mm*), rhombohedral (R, space group *R*3*c*) and the more-recently identified long-range monoclinic (M, *Cm*) phases.^8^ These distinct polar structures form different long-range ordered domains, contributing to increased domain wall densities, which are related to high dielectric permittivity and piezoelectric properties. The MPB theory complements the polarization rotation mechanism, in which the M phase serves as a structural bridge, enabling smooth polarization rotation between the R ([111]_C_) and T ([001]_C_) polar axes (where the subscript C represents the ideal cubic perovskite), thereby amplifying the piezoelectric response.^14,15^ With the discovery of the M phase in the MPB region, the Landau–Devonshire theory was extended to incorporate additional order parameters and explain the emergence of M phase, with three distinct subtypes, M_A_, M_B_ and M_C_, each defined by distinct polarization directions.^16^ Notably, M_A_ and M_B_ possess polarization vectors confined to the {110}_C_ mirror planes and are thus crystallographically indistinct within the average *Cm* symmetry. In contrast, M_C_ features polarization in the {100}_C_ planes and is assigned to the *Pm* space group.^17^ These monoclinic subphases can further enhance spatial freedom for polarization evolution. However, the MPB models inherently assume structural homogeneity at both long- and short-range scales, which oversimplifies the complex local environments in real materials. Consequently, they fall short in capturing the microscopic origins of the polarization behavior. This limitation has prompted a shift toward investigating polarization mechanisms across multiple length scales, emphasizing the interplay between long-range crystallographic order and short-range structural heterogeneity.

Since the early 2000s, growing attention has been directed toward local structural features in PZT. Neutron diffraction and pair distribution function (PDF) analysis by Dmowski et al.^18^ revealed that PZT’s real structure is significantly more complex than its average crystallographic description. Cooper et al. carried out a first-principles density functional theory (DFT) study on PZT, revealing the more ionic nature of the Zr–O bond compared to Ti–O.^19^ These early studies also established that, unlike idealized long-range ordered phases, real PZT solid solutions feature local atomic displacements that vary in both magnitude and direction. Glazer et al.^9^ used electron diffraction and revealed monoclinic ordering across varying length scales in PZT. They proposed that the long-range rhombohedral and tetragonal phases may emerge as averaged representations of a more fundamental monoclinic local structure. This perspective marked a shift in understanding the MPB – not as a sharp transition between discrete phases, but as a continuous development of local monoclinic order into extended domains. In recent years, a similar emphasis on local structural behavior emerged in relaxor ferroelectrics such as the perovskite Pb­(Me_1/3_Nb_2/3_)­O_3_–*x*PbTiO_3_ (Me = Mg, Zn, etc.), which shows ultrahigh piezoelectric performance despite its nanoscale polar order.^20^ Techniques such as diffuse X-ray/neutron scattering and PDF analysis have been widely applied to investigate such systems. Building on this foundation, Glazer and co-workers^21^ applied atomic-resolution electron microscopy to a monoclinic composition of PZT (*x* = 0.53) and revealed local disorder in B-site displacements and distortions of oxygen cages but were unable to resolve chemical heterogeneity. Further insights were provided by Zhang et al.^11^ who used neutron total scattering to probe Pb displacements in MPB compositions of PZT. On the Zr-rich side, they observed short-range M_A_ structures to coexist with long-range rhombohedral phases, while the Ti-rich compositions exhibited both M_A_ and M_C_ short-range structures.^22^ These findings indicate a fundamental duality in PZT: the long-range structure appears uniformly ordered, while the local structure has competing polar states. More recent studies have increasingly emphasized the importance of short-range structural heterogeneity in ferroelectrics.^1,23^ It is noted that previous discussions on short-range structure have mostly focused on the displacements of metallic atoms, while the role of local heterogeneity in influencing key parameters – such as remnant polarization (*P*_r_), electrostrictive coefficient (*Q*), and dielectric permittivity (ε_33_) – remains underexplored. A systematic reevaluation of this canonical system is therefore both timely and necessary. Resolving this complexity requires the integration of advanced characterization and computational tools, including atomic-resolution electron microscopy,^24,25^ DFT and molecular dynamic simulations,^26,27^ and data-driven reverse Monte Carlo modeling.^27,28^

In this work, we go beyond average space group descriptions to investigate the local structure of a prototypical MPB PZT ceramic, 0.05Pb­(Mn_1/3_Sb_2/3_)­O_3_–0.95PbZr_0.52_Ti_0.48_O_3_ (5PMS–PZT), a composition known for its structural robustness and low dielectric loss.^29–35^ By applying a comprehensive analysis across all atomic sites (A-site, B-site, and oxygen) of the perovskite structure, we uncover the fundamental polarization mechanisms to show that polarization stability and strain coherence are intimately linked to chemical ordering of the Zr/Ti cations. Moreover, we identify a previously unrecognized field-induced subphase redistribution involving variable polarization directions. These findings reflect a unity-of-opposites relationship in PZT: a seemingly single long-range phase actually emerges from multiple short-range polar states that coexist and interact. The structural stability arises from order, while the polarization flexibility arises from local disorder. Such mechanistic insights explain the exceptional polarization response characteristic of PZT and establish a generalizable concept for understanding complex structure–property relationships in high-performance piezoelectric compounds.

## 2 Results and Discussions

### 2.1 Long-Range M_A_ Phase and Its Limitations

Neutron diffraction refinements confirm that 5PMS–PZT ceramics adopt a pure monoclinic structure in both unpoled (Figure 1a) and poled (Figure S1) states. High-quality fits were achieved with low *R*-factors and minimal residuals, as shown in Table S1, while the refined atomic positions and bond geometries are summarized in Tables S2 and S3. Since this composition is in the MPB region, alternative models including tetragonal (*P*4*mm*) and rhombohedral (*R*3*m* and *R*3*c*) phases were also evaluated using Rietveld refinement. However, all other models yielded inferior fits (Figure S2). Notably, even when dual-phase models were employed, the monoclinic phase (space group *Cm*) remained dominant (>99 wt %). The presence of a single monoclinic phase is not surprising as the composition is modified from PZT (*x* = 0.48), which only shows a pure monoclinic phase at low temperatures.^36^ The monoclinic phase was independently corroborated by selected-area electron diffraction (SAED) for both unpoled (Figure 1b) and poled (Figure S3) samples. Poling did not induce a first-order phase transition but caused a small contraction of the unit cell volume by −0.25%.

**Figure 1:**
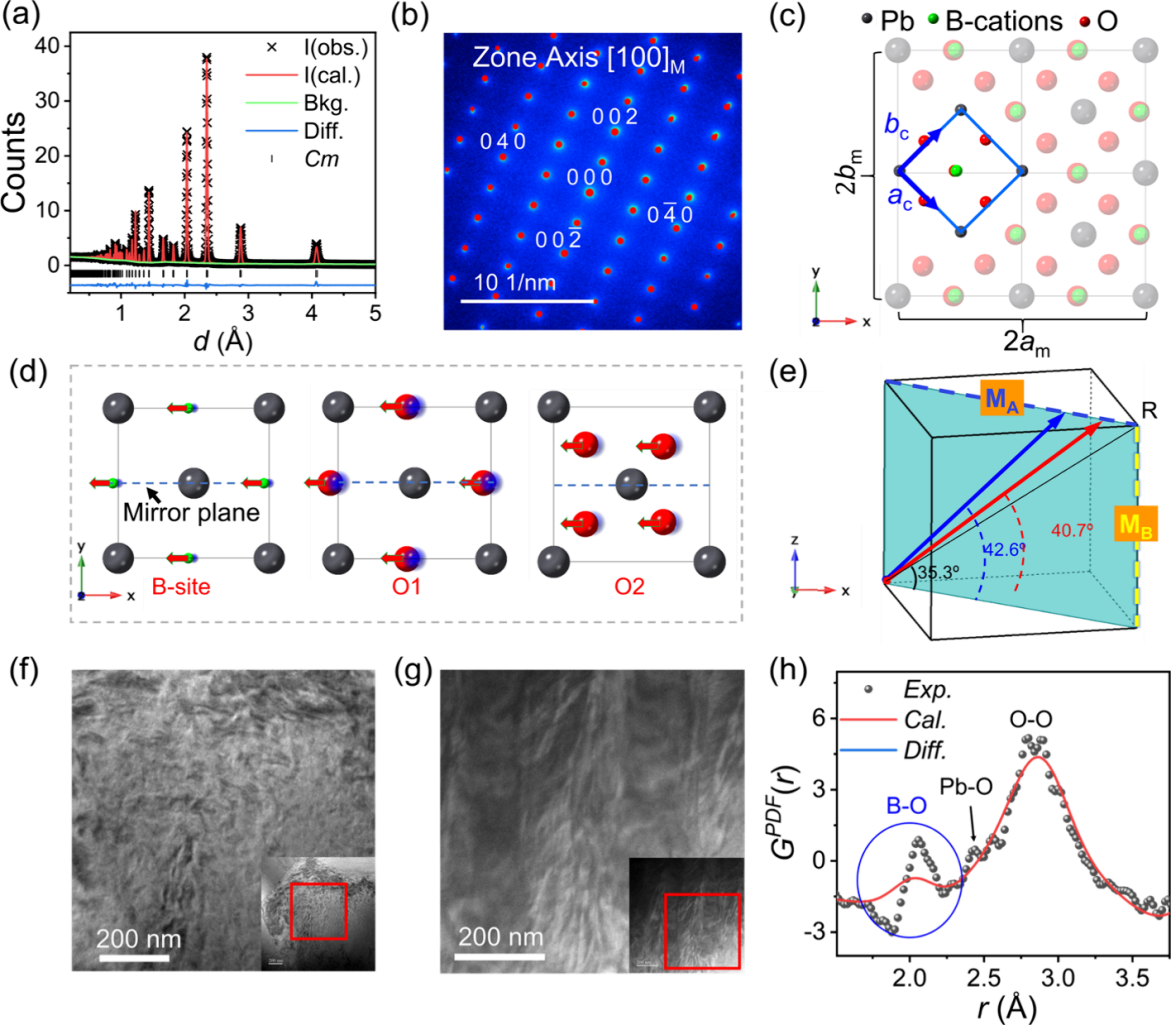
Average-structure features. (a) Fitted neutron powder diffraction pattern for the unpoled sample using the *Cm* structural model. (b) [100]_M_ zone-axis SAED pattern indexed according to the monoclinic setting. (c) Spatial relationship between the monoclinic and pseudocubic unit cells. For clarity, atoms in the *Cm* cell are faded, oxygen atoms on the top *z*-layer are omitted, and atoms in the cubic cell are shown at reduced size. (d) B-site and O atom displacements within the (110)_C_ plane, characteristic of an M_A_-type local polarization. (e) Polarization direction of 5PMS–PZT before (blue arrow) and after poling (red arrow) with respect to the equivalent pseudocubic cell. Surface domain morphology for (f) unpoled and (g) poled samples. (h) Expanded view of pair distribution function *G* ^*PDF*^(*r*) for unpoled sample fitted using the *Cm* model.

The *Cm* space group accommodates two polarization subtypes, M_A_ and M_B_, which are indistinguishable by symmetry analysis but differ in polarization orientation: the M_A_-type polarization has polarization between T and R phases with *P*_*x*_ = *P*_*y*_ < *P*_*z*_, while the M_B_-type has polarization between R and O phases with *P*_*x*_ = *P*_*y*_ > *P*_*z*_. Because ferroelectric polarization arises from displacements between the centers of positive and negative charge, quantifying atomic off-centering is critical. To facilitate this, we established a transformation matrix between the monoclinic and pseudocubic cells (Figure 1c; see also eq S1), in which the (010)_M_ mirror plane corresponds to the $(\overset{®}{1}10{)}_{C}$ plane. Atomic displacement vectors were calculated as deviations from ideal centrosymmetric positions in the pseudocubic cell, with Pb atoms fixed at the origin; thus, only B-site and oxygen displacements were considered. All displacements lie within the (010)_M_ plane (Figure 1d). Upon transformation to the pseudocubic frame, both B-site and oxygen atoms exhibit M_A_-type displacements (Table S4), resulting in an overall polarization direction at an angle of 42.6° from the [110]_C_ direction (Figure 1e). After poling, the polarization vector shifts slightly toward [110]_C_ (40.7°) and its magnitude (evaluated via eq S2) increases from 0.309 to 0.317 C m^–2^.

The thermodynamic stability of the M_A_ phase likely arises from the broader angular space (54.7°) between the tetragonal and rhombohedral polarization axes, which facilitates polarization rotation. Importantly, these results also emphasize the need for comprehensive analysis of all constituent atoms. After poling, B-site displacements have more M_B_-like character (*d*_*z*_ < *d*_*x*_), yet the net polarization remains in the M_A_ region, highlighting the critical and often overlooked contribution of oxygen displacements. Our recent studies have similarly underscored the decisive role of oxygen motion in determining net polarization in related ferroelectrics.^37,38^ Here, oxygen atoms in 5PMS–PZT display generally larger displacements than B-site cations (Figure S4a,b) and Pb–O bonds exhibit greater elongation than B–O bonds (Figure S4c), further supporting their dominant role in polarization formation.

According to the polarization rotation model,^14^ the M_A_ phase allows continuous polarization rotation within the $(\overset{®}{1}10{)}_{C}$ plane, resulting in an infinite number of possible polarization directions. Unlike in tetragonal or rhombohedral phases,^39,40^ where domain walls form along discrete, symmetry-imposed directions, M_A_-type polarization may lead to irregular domain wall configurations. This is evident in the unpoled 5PMS–PZT sample, which exhibits curved, fractal-like domain walls separated by nanosized domains (Figure 1f), in contrast to the larger strip-like domains in the poled state (Figure 1g). Such irregular domain structures are commonly seen in relaxor ferroelectrics,^41^ reflecting nanoscale polarization heterogeneity and enhanced domain wall mobility, which contribute to high dielectric permittivity. These observations suggest that 5PMS–PZT contains short-range polar features that are not constrained to fixed crystallographic planes, enabling locally minimized energies of polarization transition. The neutron difference Fourier map (Figure S5) reveals diffuse nuclear density around B-sites, indicating unresolved local structural order. However, these subtle features stretch the limits of traditional long-range structural probes like Bragg diffraction, highlighting the need for local structure analysis.

### 2.2 Breakdown of Long-Range Structure

To probe these local features, neutron pair distribution function (PDF) analysis was conducted. Fits of *G* ^PDF^(*r*) for unpoled (Figure S6a) and poled (Figure S6b) samples using the *Cm* model match well in the high-*r* region, but show significant discrepancies at low *r* (1.5 – 3.75 Å; Figures 1h and S6c), corresponding to Pb–O, B–O (B = Zr, Ti, Mn, Sb), and O–O correlations. These misfits emphasize the existence of local orders, such as chemical ordering and discrepant off-center displacements of B-cations, which are not captured by the average crystallographic model. While the *Cm* space group enforces a global mirror plane that forbids long-range out-of-plane polarization components, it permits local deviations, such as heterogeneous dipole distributions, as long as their average maintains the mirror symmetry. To capture these effects, a large-box reverse Monte Carlo (RMC) model (18150 atoms; Figure 2a) was constructed to jointly fit Bragg data, scattering function *S*(*Q*) and *G*(*r*) profiles for both unpoled- (Figure S7a,c) and poled (Figure S8a,c) samples. In the unpoled case, the model accurately reproduces the low-*r* data (Figure 2b). Despite allowing atomistic disorder, the global mirror symmetry remains preserved in the model (Figure S9), confirming that short-range structural heterogeneity coexists with long-range monoclinic order.

**Figure 2:**
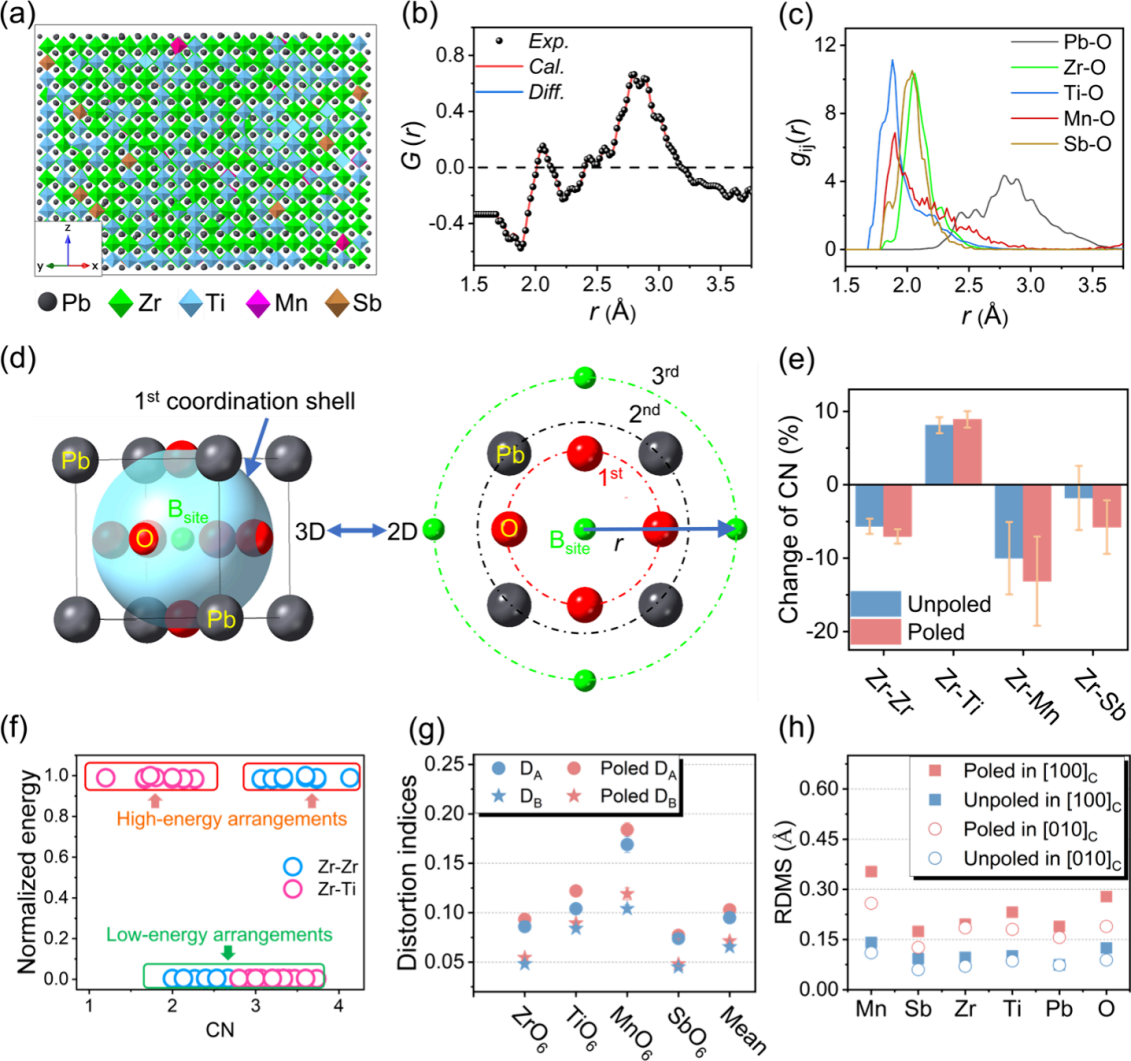
Local-structure features. (a) A representation of the RMC configuration viewed along [110]_M_. (b) Expanded view of RMC-fitted *G*(*r*) function for the unpoled sample. (c) Partial pair correlation functions for M–O pairs in the unpoled sample. (d) Schematic illustration of coordination shells around a central B-site cation in the perovskite structure, shown in both 3D view and 2D projection. (e) CN statistics for Zr in the third-nearest shell, showing reduced Zr–Zr self-coordination and a pronounced Zr–Ti mixing preference, indicative of short-range B-site chemical ordering. (f) Calculated electrostatic energy as a function of Zr CN. (g) Angle and bond distortion indices (*D*_A_ and *D*_B_) of BO_6_ octahedra in unpoled and poled structures. (h) Root-mean-square displacements (RMSD) of atomic positions in unpoled and poled samples, showing stronger local off-centering of atoms after poling.

### 2.3 Correlation of Chemical Heterogeneity and Polarization Stability

Partial pair distribution functions (PDFs) derived from both unpoled (Figure 2c) and poled (Figure S10a) structures reveal asymmetric Pb–O peaks featuring two distinct maxima centered at ∼2.5 and ∼2.8 Å. These correspond well with the average bond lengths obtained from long-range analysis (Table S3) and are consistent with prior reports on PZT.^11,23^ Significant variation is observed in the B–O correlations: Zr–O bonds are the longest, while Ti–O bonds are the shortest. The high contrast in neutron scattering lengths between Zr and Ti (Table S5), enhances spatial resolution and allows confident differentiation of local bonding environments. The extended Zr–O bond length is in line with the larger ionic radius of Zr^4+^ (Table S6) and consistent with the findings of Cooper et al.^19^ and the results obtained from EXAFS.^42^ In addition, Zr–O and Sb–O show sharper, more symmetric profiles, whereas Ti–O and Mn–O peaks are broader and asymmetrically skewed, indicating greater distortions in TiO_6_ and MnO_6_ octahedra. These distortions are often associated with higher piezoelectric activity.^11^ The averaged M-O bond lengths derived from the RMC model are consistent with the DFT predictions (Table S5).

To quantify local coordination, we calculated coordination numbers (CNs) from the partial PDFs using eq S3 (schematically shown in Figure 2d). The calculated CNs for Pb (12) and B-site cations (6) agree well with theoretical expectations (Figure S10b), confirming the integrity of the PbO_12_ and BO_6_ polyhedra and suggesting minimal oxygen vacancies. To investigate chemical ordering on the B site, we examined the third-nearest-shell CN of Zr and Ti, which dominate the B-site occupancy. We compared the experimental CNs with theoretical values predicted for a fully random B-site distribution, calculated based on stoichiometry via eq S4. The results obtained in the final configurations, expressed as percentages relative to the random distribution, are shown in Figure 2e (Zr) and Figure S11a (Ti). Notably, Zr exhibits 5.6% fewer Zr–Zr self-pairs and 8.1% more Zr–Ti pairs than expected from a random distribution. A similar trend was found for Ti, which is a clear indication of antiself-clustering behavior. This is the first direct evidence of anticlustering behavior in PZT, prompting an investigation into its energetic origin. Electrostatic energies were evaluated across >10^12^ supercell configurations with varying Zr–Ti distributions. As shown in Figures 2f and S11b, structures with well-mixed Zr–Ti coordination consistently display lower electrostatic energies than those with Zr–Zr or Ti–Ti clustering. These results suggest that antiself-clustering is energetically favored, offering a thermally stable polar state once it is formed, which contributes to the high remnant polarization (*P*_r_).

Chemical heterogeneity also directly influences local lattice distortions. The BO_6_ octahedral distortion indices, *D*_A_ (angular) and *D*_B_ (bond length), as defined by eqs S5 and S6,^43^ reveal that TiO_6_ and MnO_6_ units are inherently more distorted than ZrO_6_ and SbO_6_ (Table S6). This is attributed to the higher covalency of Ti–O bonds, which enables greater bond flexibility, in contrast to the more ionic and rigid Zr–O bonds—a trend consistent with previous DFT studies.^19^ The ionicity of a chemical bond can be evaluated by using eq S7, with the results showing 67 and 59% for Zr–O and Ti–O, respectively. Given that Zr and Ti are the dominant B-site cations, their associated octahedra primarily shape the overall local structural distortion (Table S6). DFT-based force perturbation calculations further reveal that displacing Zr atoms requires higher restoring forces (0.650 eV/Å) than Ti atoms (0.611 eV/Å), indicating that Zr-centered environments are more rigid, whereas Ti-centered environments are softer and more adaptable. Thus, introducing Ti-rich local environments around Zr enables more compliant bonding configurations, which helps accommodate local mechanical stress. This stress relaxation enhances polyhedral flexibility and thereby strengthen the coupling effect between polarization and strain, contributing to a high electrostriction coefficient (*Q*).

Collectively, these findings provide strong evidence that short-range Zr–Ti mixing not only reduces electrostatic energy but also alleviates internal stress, thereby stabilizing multivariant polarization states within the monoclinic phase. After poling, the increased distortion indices across all BO_6_ units (Figure 2g) and higher atomic root-mean-square displacement (RMSD, Figure 2h, defined by eq S8) suggest further mismatches between charge centers. Notably, significant displacements are observed not only for B-site cations, but also for Pb and O atoms, highlighting the necessity of a comprehensive, all-atom structural analysis when investigating polarization mechanisms.

### 2.4 Short-Range Atomic Off-Center Displacements and Polarization Structures

The displacement of individual atoms in the final RMC configurations relative to their ideal centrosymmetric positions were examined and are plotted as displacement vector density projections in Figure 3a for 5PMS–PZT in the unpoled state, with those for the poled state given in Figure S12. Projections onto the (010)_M_ plane reveal that Pb atoms in the unpoled state predominantly displace along the [101]_M_ direction, albeit with a high degree of positional disorder. B-site cations exhibit different displacement tendencies in the [101]_M_ direction depending on the type of cation, with Ti and Mn displaced in the same direction as Pb, while Zr and Sb are displaced in the opposite direction. Interestingly, the displacement of O atoms in the [101]_M_ direction are coherent with those of Zr and Sb, consistent with the lower distortion levels observed in ZrO_6_ and SbO_6_ octahedra. Projection onto the (001)_M_ plane (Figure 3a) shows displacement densities that are fairly symmetric with respect to the (010)_M_ mirror plane corresponding to the horizontal dashed line in each projection. Significant displacements are seen in the [100]_M_ direction, with Pb and Mn displaced in one direction and Zr, Sb and O displaced in the opposite direction. Interestingly, Ti shows little evidence of displacement in the [100]_M_ direction. For the poled structure (Figure S12), projections reveal similar displacement distributions compared to the unpoled state, with the main difference being a more evident displacement of Ti in the [100]_M_ direction.

**Figure 3:**
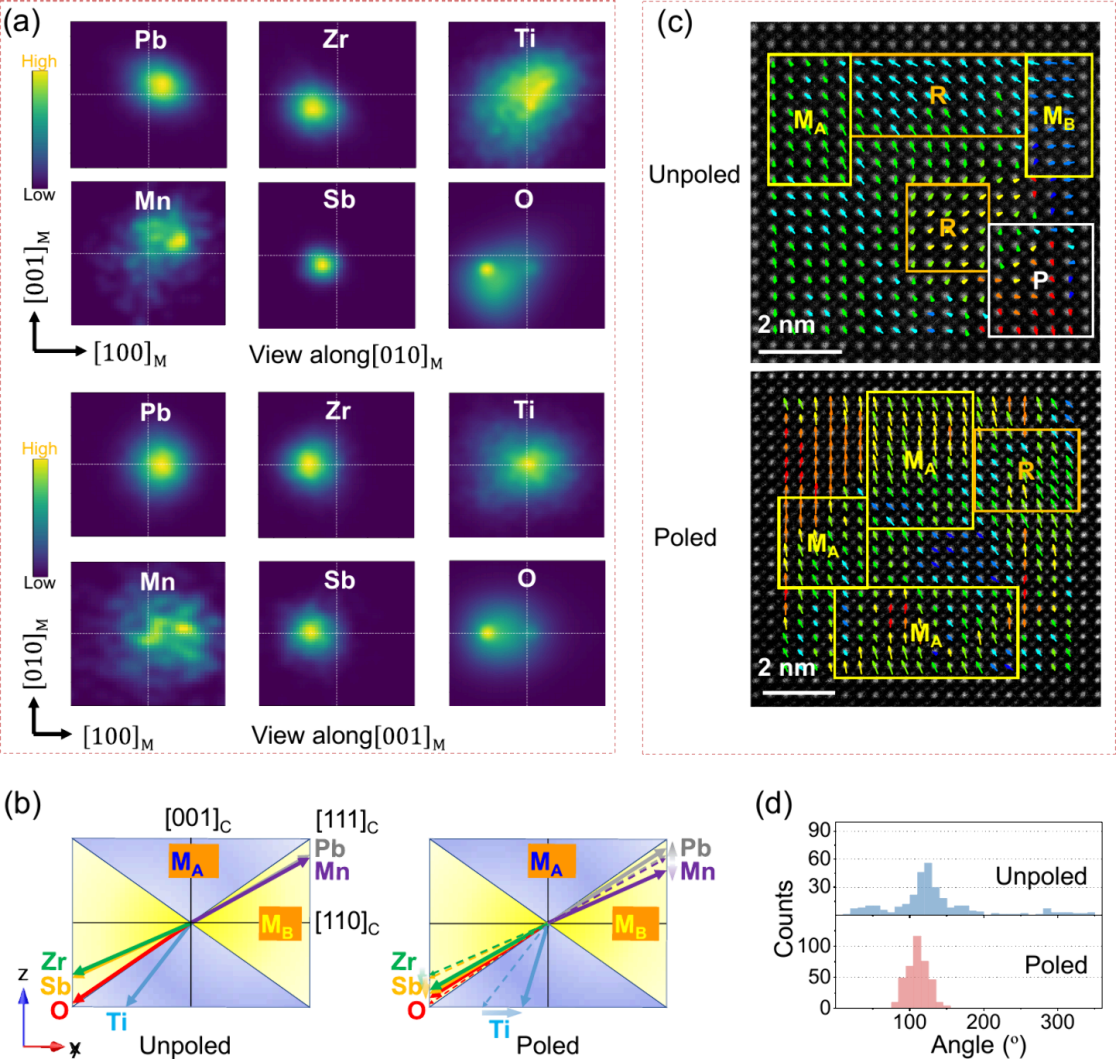
Atomic off-center displacements. (a) Projected density map of atomic off-center displacement vectors along [010]_M_ and [001]_M_ directions. (b) Averaged displacement direction of individual atom types in the equivalent pseudocubic unit cell in both unpoled and poled states (viewed along $[\overset{®}{1}10{]}_{C}$ ). (c) HRTEM image of Pb atoms (bright spots) relative to the centroids of adjacent B-site atoms (weaker spots), with Pb displacement vectors represented by arrows. The arrow colors correspond to the displacement magnitudes, ranging from low (green) to high (red) values. (d) Angular histograms of Pb displacement directions with respect to the [100]_C_ axis.

The averaged atomic displacement vectors are illustrated in (Figure 3b). Interestingly, while the vector density projections in Figure 3a appear to show Ti displacement in the same direction as Mn, when averaged over all Ti atoms in the model, the overall displacement is in the opposite direction. There are subtle directional differences among the B-site atoms: The overall Ti displacement vector lies within the M_A_-type orientation field (indicated by the blue color in Figure 3b), while the Zr, Mn, and Sb displacement vectors lie within the M_B_-type orientation field (yellow color) in the unpoled sample. The behavior of Ti is consistent with prior observations in PZT.^44^ Oxygen displacement is in the [111]_C_ direction, corresponding to a rhombohedral type displacement in the equivalent cubic perovskite cell. Upon poling, the average displacement vectors reorient somewhat: Ti vectors rotate toward the tetragonal [001] axis, being similar to previous finding in another PZT composition (*x* = 0.50),^44^ while Pb, Zr, and Sb align closer to the rhombohedral [111]_C_ direction. The enhanced rotation of the Ti vector reflects the greater spatial freedom afforded by the M_A_ region. Further vector density projections onto the (111)_C_ plane (Figure S13a for unpoled and Figure S13b for poled states) confirm that Pb, Zr, Sb show little displacement around [111]_C_, while Ti and Mn show significantly higher levels of disorder around (111)_C_ although they remain fairly symmetrically displaced. A similar observation for Zr and Ti displacements was also reported by Dmowski et al.^18^

The different atomic displacive directions support the formation of complex short-range polar regions. Such short-range structural features likely contribute to the irregular morphology of domain walls, which may facilitate enhanced polarization rotation. These behaviors are visualized in atomic-resolution TEM images (Figure 3c), where the localized Pb and B-site column displacements reveal the apparent coexistence of M-, R- and P-type (pseudocubic) substructures. The apparent existence of a P-type substructure should be interpreted cautiously since the image could simply occur from the superposition of M- and R- type substructures when viewed in projection. Indeed, on poling, P-type regions are no longer observed, while the Pb displacements becoming more coherent with a predominance of M_A_- and R-type orientations. This realignment is quantitatively supported by the narrower angular distribution of Pb displacement vectors (Figure 3d).

While prior studies defined local polarization based only on Pb displacement relative to its oxygen cage centroid,^11^ our data in Sections 2.1 and 2.4 show that both B-site and oxygen atoms contribute significantly to the local polar structure. Thus, we calculated the polarization vector of each crystallographic subcell in the RMC supercell, taking all atomic species into account. To aid analysis, the polarization vectors were transformed to the pseudocubic set by using the conversion matrix (eq S1) and were then projected onto a spherical surface (Figure 4a). This allows clear identification of the characteristic polarization directions along tetragonal ([001]_C_, [100]_C_ or [010]_C_), orthorhombic ([110]_C_, [101]_C_ or [011]_C_) and rhombohedral (along [111]_C_) polar axes. In this way, the monoclinic region remains within the mirror planes which are defined by tetragonal and orthorhombic axes, i.e., the $(1\overset{®}{1}0{)}_{C}$ plane defined by [001]_C_ and [110]_C_, the $(\overset{®}{1}01{)}_{C}$ plane by [101]_C_ and [010]_C_, and the $(0\overset{®}{1}1{)}_{C}$ plane by [011]_C_ and [100]_C_. These mirror planes with different normal directions originate from differently oriented monoclinic domains. The M_A_ (highlighted by blue lines) and M_B_ (yellow lines) regions are also easily distinguished.

**Figure 4:**
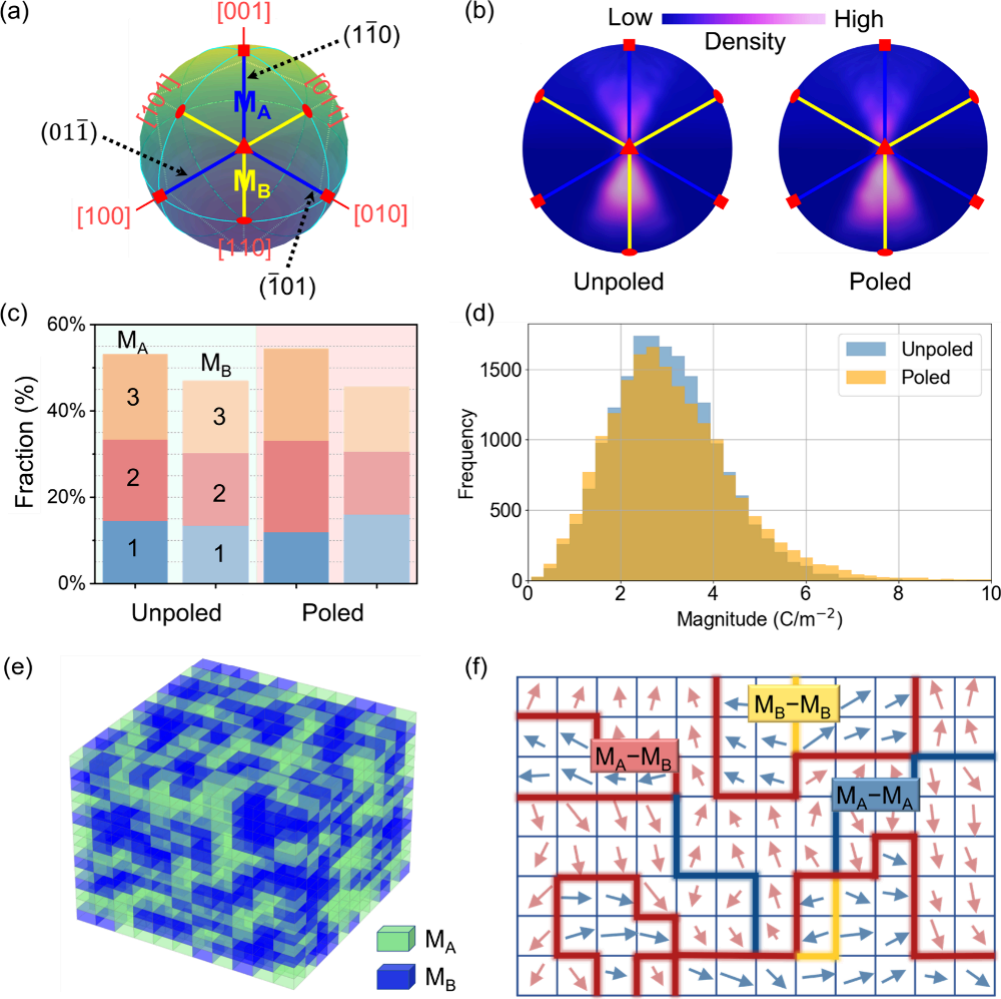
Polarization and local domain structures. (a) Schematic spherical projection method. Crystallographic axes [100]_C_, [010]_C_, and [001]_C_ are extended from the unit cell center to the sphere’s surface to aid visualization of polarization orientations. (b) Spherical projection of local dipole directions in unpoled and poled structures. (c) Monoclinic subphase populations (M_A_ and M_B_) related to mirror planes (1 for $(1\overset{®}{1}0{)}_{C}$ , 2 for $(\overset{®}{1}01{)}_{C}$ , and 3 for $(0\overset{®}{1}1{)}_{C}$ ). (d) Frequency of polarization magnitudes for sub cells in the RMC configurations, derived from ten independent models. (e) Spatial distribution of local M_A_ (green) and M_B_-type (blue) domains in a representative RMC model. (f) A schematic map showing three different types of irregular domain walls.

In this way, the polarization vectors were separated into three groups according to their nearest mirror plane, and then projected onto the spherical surface. The first group ( $(1\overset{®}{1}0{)}_{C}$ associated) reveals higher density in the M_B_ region suggesting the polarization directions in this domain are more coherently aligned. In contrast, the other two groups (associated with $(\overset{®}{1}01{)}_{C}$ and $(0\overset{®}{1}1{)}_{C}$ ) show higher vector densities within the M_A_ region (Figure S14), underscoring the coexistence of both M_A_ and M_B_-type polarization structures on a local scale. This observation contrasts with the long-range structure, which predominantly exhibits a typical M_A_-type polarization state (Figure 1e). The coplanar distribution of these local polarization vectors facilitates smooth polarization rotation paths, thereby enhancing dielectric permittivity and contributing to higher piezoelectric performance.

Statistical analysis of M_A_ and M_B_-type subphases in the three different groups reveals a change after poling indicative of local phase transitions between M_A_ and M_B_ substructures under an applied electric field (Figure 4c), while preserving the overall *Cm* symmetry. The findings show that the high mobility and configurational flexibility of local polar structures allows transitions across intersecting mirror planes, as a result, high mobility of domain walls is enabled, associated with high permittivity. After poling, the polarization magnitude of vectors aligned with the electric field direction increased, whereas those oriented in the opposite direction diminished in intensity (Figure 4d), reflecting a field-induced reconfiguration of local polarization vectors.

## 3 Mechanisms for Enhanced Piezoelectricity at MPB

The coexistence of multiple short-range M_A_ and M_B_-type polar structures offer new insights into the polarization behavior of PZT-based piezoelectrics near the MPB. While the long-range structure exhibits a monoclinic M_A_-type phase, it can be viewed as an average over locally distinct M_A_ and M_B_ subphases. This nanoscale polarization heterogeneity contributes to a complex domain structure, as evidenced by the RMC model (Figure 4e), which clearly resolves M_A_ (green) and M_B_ (blue) domains extending across just a few nanometers. Such short-range ordering provides a structural origin for the fractal-like domain wall morphologies observed via TEM in Figure 3d. Consequently, three types of domain walls, M_A_–M_A_, M_A_–M_B_ and M_B_–M_B_, can form, each exhibiting irregular and diffuse boundaries (Figure 4f).

These findings establish a comprehensive relationship between local structural heterogeneity and macroscopic piezoelectric performance in high-performance PZT ceramics with MPB compositions (Figure 5). The presence of mixed short-range polar domains (M_A_ and M_B_) introduces continuous regions of accessible polarization directions, effectively lowering the energy barrier for polarization rotation to enable high remnant polarization. This also facilitates local dipole reorientation and enhances dielectric permittivity. The chemical heterogeneity at the B-site contributes to a locally coherent BO_6_ framework (TiO_6_ and ZrO_6_) that accommodates strain, resulting in a high electrostriction coefficient and stabilization of polar configurations, leading to high remnant polarization after poling. Together, these effects synergistically promote superior piezoelectric responses in PZT near the MPB. Importantly, our findings emphasize the limitations of relying solely on average symmetry descriptions to understand piezoelectric behavior. Instead, comprehensive models must account for both structural and chemical heterogeneities—such as cation disorder, atomic displacement, spatial polarization distributions, octahedral tilts and distortions, and lattice strain, all of which significantly impact dielectric behavior, electrostriction and remnant polarization, enabling the superior piezoelectric properties of PZT.

**Figure 5:**
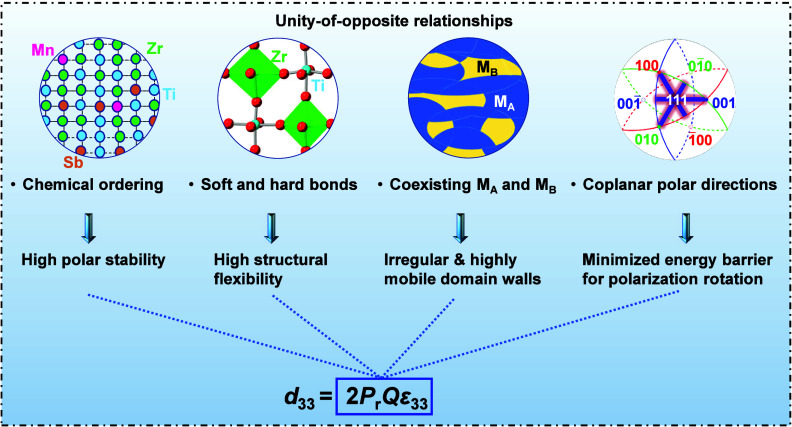
Mechanisms for enhanced piezoelectric performance in PZT ceramic near the MPB.

## 4 Conclusions

In this work, we reveal that the extraordinary piezoelectric performance, associated with polarization response of perovskite PZT near the MPB emerges from a set of unified chemical opposites: rigid and soft bonds, long-range order and local disorder, distinct polar states and coplanar polarization directions.

First, the seemingly incompatible soft and hard chemical bonds self-organize through B-site chemical ordering to form a coherent BO_6_ network. This arrangement helps minimize local mechanical stress and Coulombic energy, contributing to intrinsically high stability of polar state and supporting a high remnant polarization (*P*_r_) once a new polar state is induced. Simultaneously, this soft–hard compatible bonding network enhances octahedral flexibility, strengthening polarization-strain coupling and thereby boosting the electrostriction coefficient (*Q*).

Second, a long-range ordering monoclinic phase with embedded short-range M_A_ and M_B_-type polar states together shape the nanoscale domains with irregular and highly mobile domain walls, contributing to a high dielectric permittivity (ε_33_). These multiscale polar states also maintain a polar configuration with high thermal stability.

Third, the distinct M_A_ and M_B_ polar states, associated with directionally different off-center displacements of A-site, B-site and O atoms, have coplanar polar directions, which minimize the energy barrier for ferroelectric polarization rotation and further enhance the dielectric permittivity and piezoelectric response.

Together, these factors do not compete but cooperate, creating a balanced yet highly adaptable polar lattice. By moving beyond average symmetry to investigate real-space structural heterogeneity, we find the chemical origin of high piezoelectricity of the PZT oxides, and show how the complex unity-of-opposite relationships govern polarization behavior in this canonical system. This duality-based analysis should be broadly applicable to complex perovskites and it offers guiding principles for the design of high-performance dielectric, piezoelectric and ferroelectric materials.

## Supplementary Material



## Data Availability

The data that support the findings of this study are available from the corresponding author upon reasonable request.

## Notes

Materials preparation (powder and ceramic samples of 0.05Pb­(Mn_1/3_Sb_2/3_)­O_3_–0.95Pb­(Zr_0.52_Ti_0.48_)­O_3_); characterization (neutron total scattering and transmission electron microscopy); data analysis (Rietveld refinements, pair distribution function (PDF) analysis, Coulombic energy calculations, and DFT calculations) (PDF)
