# Supplementary material for: Structural Origin of Morphotropic Phase Boundary in Advanced Perovskite Ferroelectric Oxides
Source: J Am Chem Soc. 2026 Feb 13;148(7):7827–36. doi: 10.1021/jacs.5c22401 (PMC12951426; doi:10.1021/jacs.5c22401)
Supplement: Supplementary file 1 [file ja5c22401_si_001.pdf]

Supporting Information for

Structural Origin of Morphotropic Phase Boundary in Advanced Perovskite Ferroelectric Oxides

Yajun Yue[a*#], Fengjin Qu[b,h#], Giuseppe Viola[c], Bing Han[d], Marcin Krynski[e], Takashi Honda[f], Qifeng Zheng[a*], Zimeng Hu[d*], Isaac Abrahams[g], Haixue Yan[c*]

[a] School of Chemistry, South China Normal University, Guangzhou 510006, Guangdong, China.

[b] Spallation Neutron Source Science Center, China Spallation Neutron Source, Dongguan 523803, Guangdong, China.

[c] School of Engineering and Materials Science, Queen Mary University of London, Mile End Road, London E1 4NS, UK.

[d] Key Laboratory of Inorganic Functional Materials and Devices, Shanghai Institute of Ceramics, Chinese Academy of Sciences, 588 Heshuo Road, Jiading, Shanghai 201899, People's Republic of China.

[e] Faculty of Physics, Warsaw University of Technology, ul. Koszykowa 75, 00-662 Warsaw, Poland.

[f] J-PARC Center, High Energy Accelerator Research Organization (KEK), Tokai, Ibaraki 319-1106, Japan.

[g] Department of Chemistry, Queen Mary University of London, Mile End Road, London, E1 4NS, U.K.

[h] Institute of High Energy Physics, Chinese Academy of Sciences (CAS), Beijing 100049, China.

[#] These authors contributed equally to this work and should be considered as co-first authors.

Contents

1. Experimental methods 4

1.1 Materials preparation 4

1.2 Characterization 4

1.2.1 Neutron Total Scattering 4

1.2.2 Transmission Electron Microscopy (TEM) 4

1.3 Data analysis **5**

1.3.1 Rietveld Refinements 5

1.3.2 Pair Distribution Function (PDF) Analysis 5

1.3.3 Coulombic energy calculations 6

1.3.4 DFT Calculations 6

Figures 7

Fig. S1 7

Fig. S2 8

Fig. S3 9

Fig. S4. 10

Fig. S5 11

Fig. S6 12

Fig. S7. 13

Fig. S8 14

Fig. S9 15

Fig. S10 16

Fig. S11 17

Fig. 12 18

Fig. S13 19

Fig. S14 20

Tables 21

Table S1. 21

Table S2. 22

Table S3. 23

Table S4. 24

Table S5. 25

Table S6. 26
Table S7. 27
Table S8. 28
Equation S1:..29
Equation S2:..30
Equation S3:..31
Equation S4:..32
Equations S5 and S6:..33
Equation S7:..34
Equation S8:..35
Equation S9:..36
References **..37**

1. Experimental methods

1.1 Materials preparation

Samples of composition $0.05Pb(Mn_{1/3}Sb_{2/3})O_3$–$0.95Pb(Zr_{0.52}Ti_{0.48})O_3$ were prepared through a conventional solid-state reaction method. For each sample, stoichiometric amounts of PbO (Sigma, 99.9%), Mn_3O_4 (Sigma, 99%), Sb_2O_3 (Sigma, 99.99%), ZrO_2 (Sigma, 99%) and TiO_2 (Sigma, 99.8%) were pre-heated at 200 °C overnight and then milled together in ethanol for 6 h using Y-doped ZrO_2 balls in a planetary ball mill (P5 model IV, Nanjing Machine Factory, China). The powder was then dried, sieved and calcined in an air furnace (Nabertherm, UK) at 800 °C for 2 h followed by 850 °C for 2 h. After cooling, the powder was ball-milled for another 6 h and mixed with 7 wt% polyvinyl alcohol (PVA) as a binder. Green pellets were prepared by pressing the powders in a mold (diameter = 13 mm, thickness ≈ 0.5 mm) under a uniaxial pressure of 150 MPa, followed by heating at 650 °C for 2 h to remove the binder and subsequently at 1250 °C for 2 h for sintering. Pellets were then ground to ca. 6 mm in diameter and polished. For poling, Ag paste electrodes were applied and samples poled under a direct current field of E = 4.5 kV/mm for 15 min in silicone oil.

1.2 Characterization

1.2.1 Neutron Total Scattering

Neutron total scattering experiments were performed at room temperature on the NOVA diffractometer at the Japan Proton Accelerator Research Complex (J-PARC). The unpoled and poled pellets were then placed in a V-Ni alloy cell of 6 mm diameter. Scans were carried out for ca. 6 hours and the data from the 90° bank were corrected by subtracting the intensities from the V-Ni alloy cell and normalized to the incident beam with data for a 6 mm diameter V-rod.

1.2.2 Transmission Electron Microscopy (TEM)

Before TEM measurements, both unpoled and poled 5PMS-PZT pellets were polished to 30 μm in thickness using an Ar^+ ion-milling Gatan PIPS II system (Gatan Inc., Pleasanton, CA). Specimens were then coated with a thin carbon layer (ca. 3 nm in thickness) to minimize charging

effects by the electron beam. Bright field images and selected area electron diffraction (SAED) patterns were collected using a JEOL JEM-2100F microscope equipped with a charge-coupled device camera. Atomic-scale high-angle annular dark-field (HAADF) images were collected under a Cs-corrected Hitachi HF5000 microscope, with the probe fitted in UHR mode, a convergence semi-angle of 20 mrad, and a collection semi-angle of 60 - 320 mrad.

Direct images were captured using a fast-scan mode and averaged over multiple frames. It is noted that although samples were imaged at room temperature, it is estimated that they were heated to around 45~65 °C by the electron beam. This heating does not induce a substantial depoling of the specimens as their Curie points are at much higher temperatures. The precise atomic column positions with picometer precision were extracted and refined using a least-squares fitting method[1-2]. The off-center displacements of Pb atoms were calculated with respect to the centroid of the neighboring B site cations, as reported previously[3].

1.3 Data analysis

1.3.1 Rietveld Refinements

Neutron powder diffraction data were analyzed using Rietveld refinement with the GSAS suite of programs via EXPGUI[4-5]. Several models were tested in refinements, including monoclinic (*Cm*, a = 5.708, b = 5.707, c = 4.141 Å and β = 90.199°), tetragonal (*P*4*mm*, a = 4.055 and c = 4.108 Å) and rhombohedral (*R*3*m*, a = 4.07 Å and = 89.65°) structures[6-8].

1.3.2 Pair Distribution Function (PDF) Analysis

The corrected and normalized scattering data obtained from neutron total scattering experiments were further processed by using the Stog script implemented in the RMCprofile software[9] to obtain the structure function $S(Q)$, the radial distribution function $D(r)$ and the total pair distribution function $G(r)$. The average model (*Cm*) was then tested in the PDFgui software[10] using the modified $G^{PDF}(r)$ function. The relationships of the correlation functions used in this work can be found in Equation S9 in the supporting material.

Large-box modelling was performed using the RMCprofile software with the reverse Monte

Carlo (RMC) method to fit the total scattering datasets. The neutron Bragg data were used as a constraint to maintain long-range ordering. For each sample, 10 initial models were built with random distributions of B-site atoms in 11 × 11 × 15 supercells of the corresponding average *Cm* unit cell, but with idealized atomic coordinates corresponding to a centrosymmetric *C*2/*m* model (i.e. non-polar). In modelling, soft bond valence summation (BVS) constraints were applied, with the bond-stretching pseudopotential constraints applied on metal-oxygen pairs to maintain realistic bond length distributions. B-site atoms were allowed to swap positions during calculations. Calculations were performed for 8 days and the results obtained were averaged over the ten final configurations.

1.3.3 Coulombic energy calculations

Local occupational ordering of the B-site cations was further evaluated by calculating the Coulombic energy for ca. 1.2×10^{12} supercells of size 2 × 2 × 4 with respect to the initial *Cm* model using the Supercell program[11]. All possible arrangements of B-site cations were examined, with 600 models selected according to energy from high to low.

1.3.4 DFT Calculations

Density functional theory (DFT) calculations were carried out using the Vienna Ab initio Simulation Package (VASP)[12] with the projector-augmented wave (PAW) method[13]. To avoid the overestimation of electron delocalization and accurately describe the electronic structure of transition metal elements, the Strongly Constrained and Appropriately Normed functional (SCAN)[14] together with the Dudarev[15] approach of the Hubbard U correction (DFT+U) were employed in calculations. The U parameters were set to 2 eV and 4 eV for Zr and Ti, respectively. A plane-wave cutoff energy of 450 eV was used to ensure convergence, and Brillouin zone integration was performed at the gamma point. Structural relaxations were conducted until the forces on all atoms were below 10^{-5} eV/Å. To model the effects of doping and vacancies, supercells of 270 atoms were constructed. All the results presented were obtained from relaxed structures

Figures

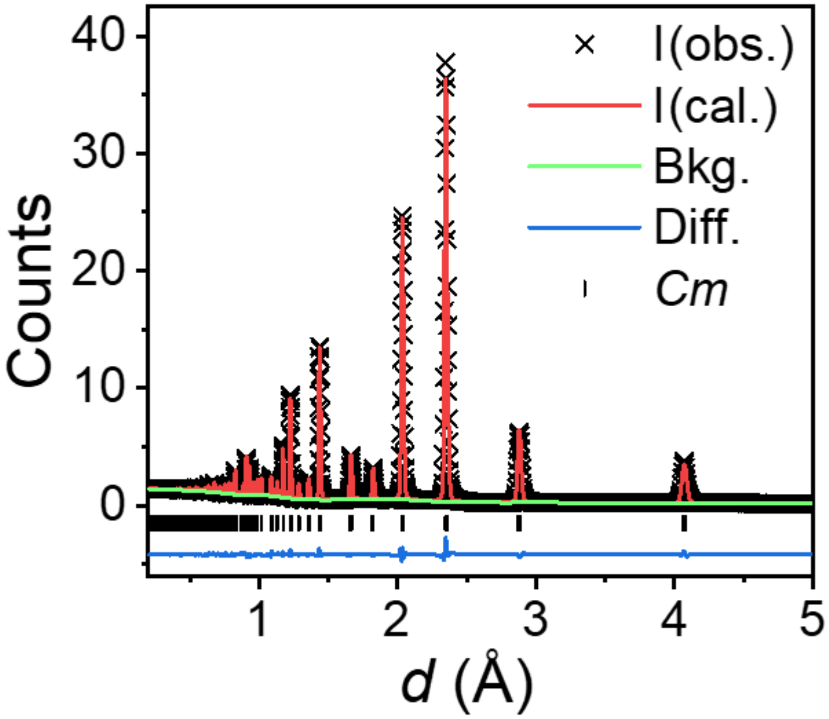

Fig. S1 Fitted neutron powder diffraction patterns for poled 5PMZ-PZT using the *Cm* model.

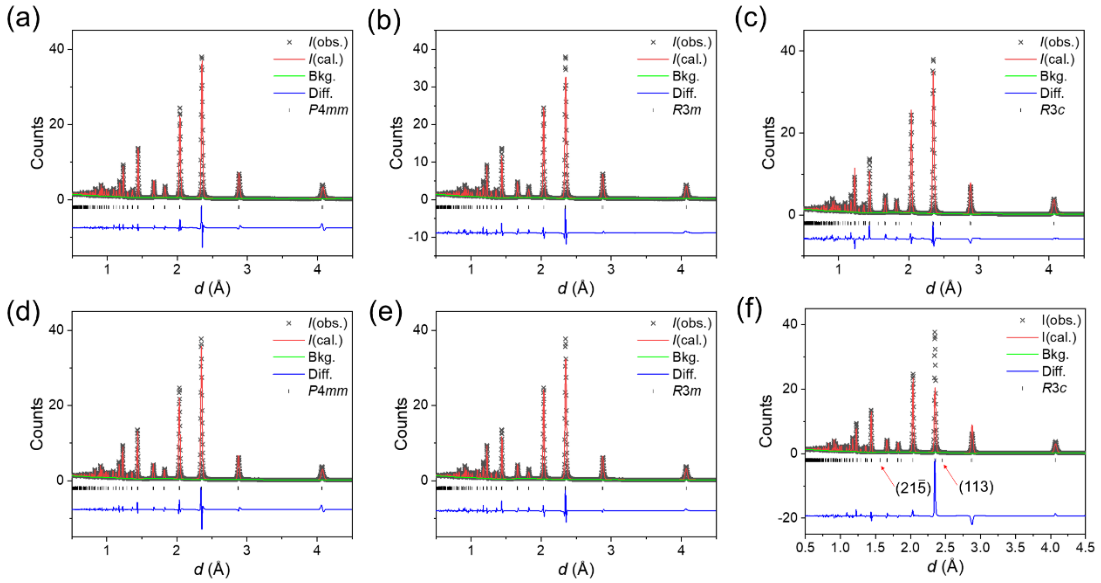

Fig. S2 Attempted fittings to neutron powder diffraction patterns for (a-c) unpoled and (d-f) poled 5PMZ-PZT using (a, d) *P*4*mm*, (b, e) *R*3*m* and (c, f) *R*3*c* models.

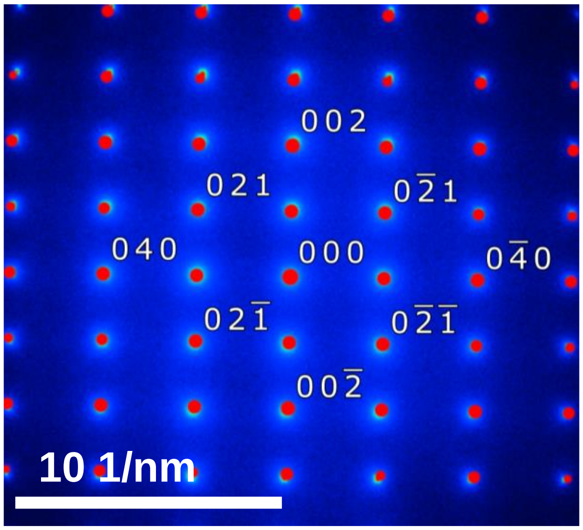

Zone Axis [100]

Fig. S3 The $[100]_M$ zone-axis selected area electron diffraction (SAED) pattern for poled 5PMS-PZT indexed according to the monoclinic setting.

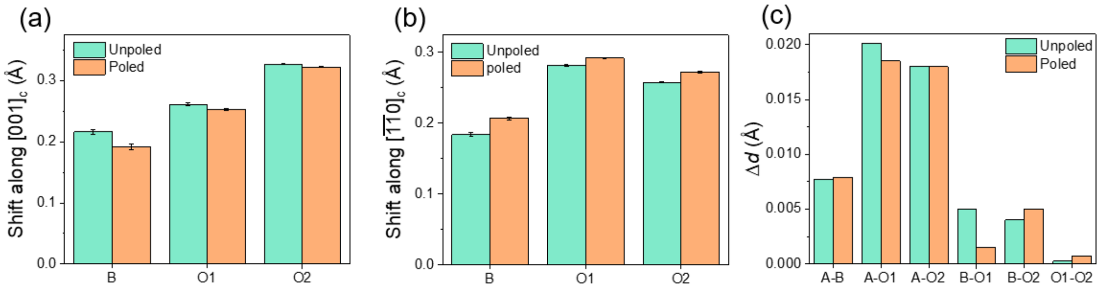

Fig. S4. (a) and (b) atomic shifts from their ideal sites along (a) $[001]_C$ direction and (b) $[\bar{1}10]_C$ direction. (c) The variation of bond lengths in the monoclinic cell compared to those obtained in an ideal centrosymmetric cell.

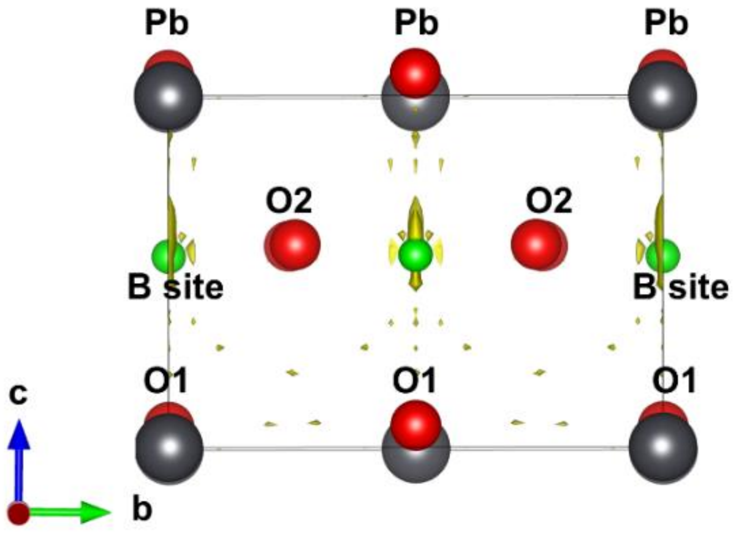

Fig. S5 Difference Fourier map showing disordered B site in the *Cm* model (iso-surface level = 0.33). For clarity, B-site cations are reduced in size.

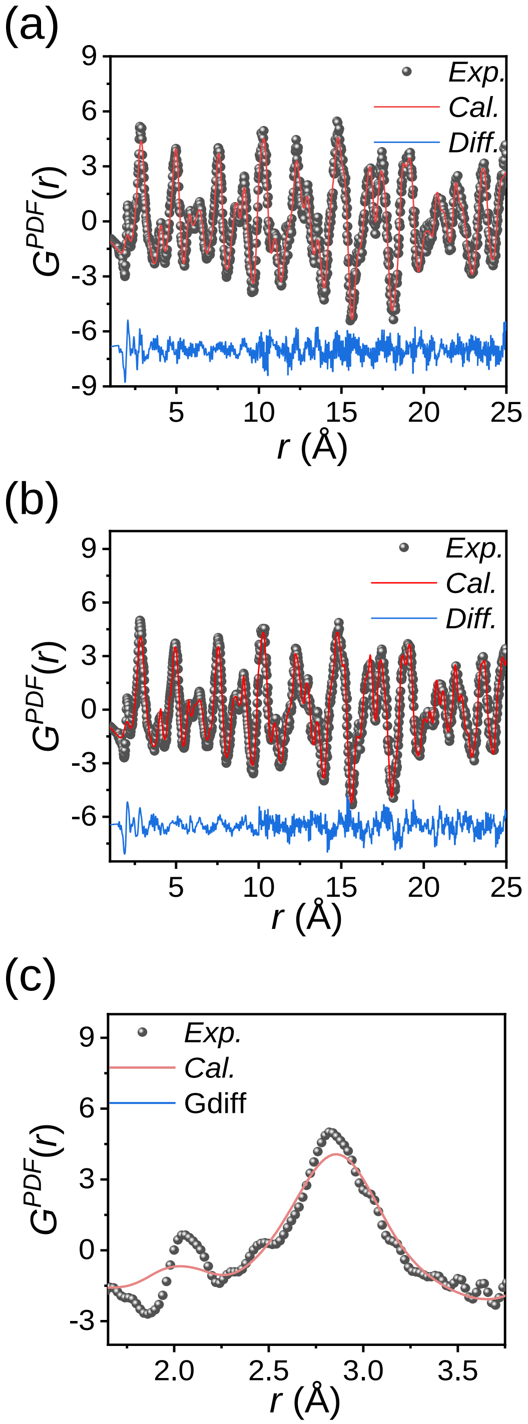

Fig. S6 (a) and (b) fitted $G^{\mathrm{PDF}}(r)$ profiles using the *Cm* model for (a) unpoled and (b) poled 5PMS-PZT, with (c) showing the expanded view of (b). The red and green lines in (c) represent calculated profiles using the *Cm* model without and with consideration of B-site ordering.

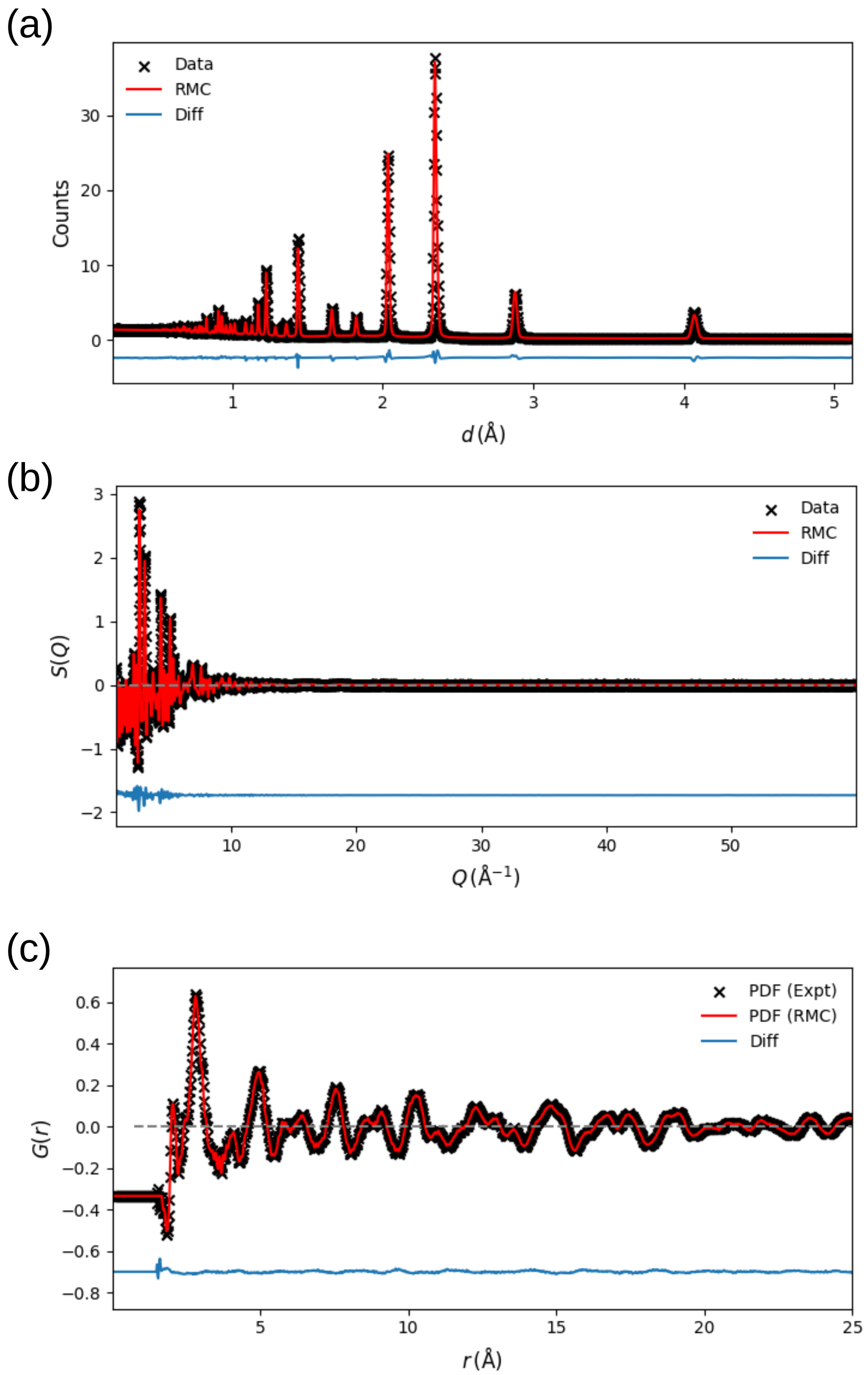

Fig. S7. Fitted (a) Bragg (b) $S(Q)$ and (c) $G(r)$ data for unpoled 5PMS-PZT using the RMC method.

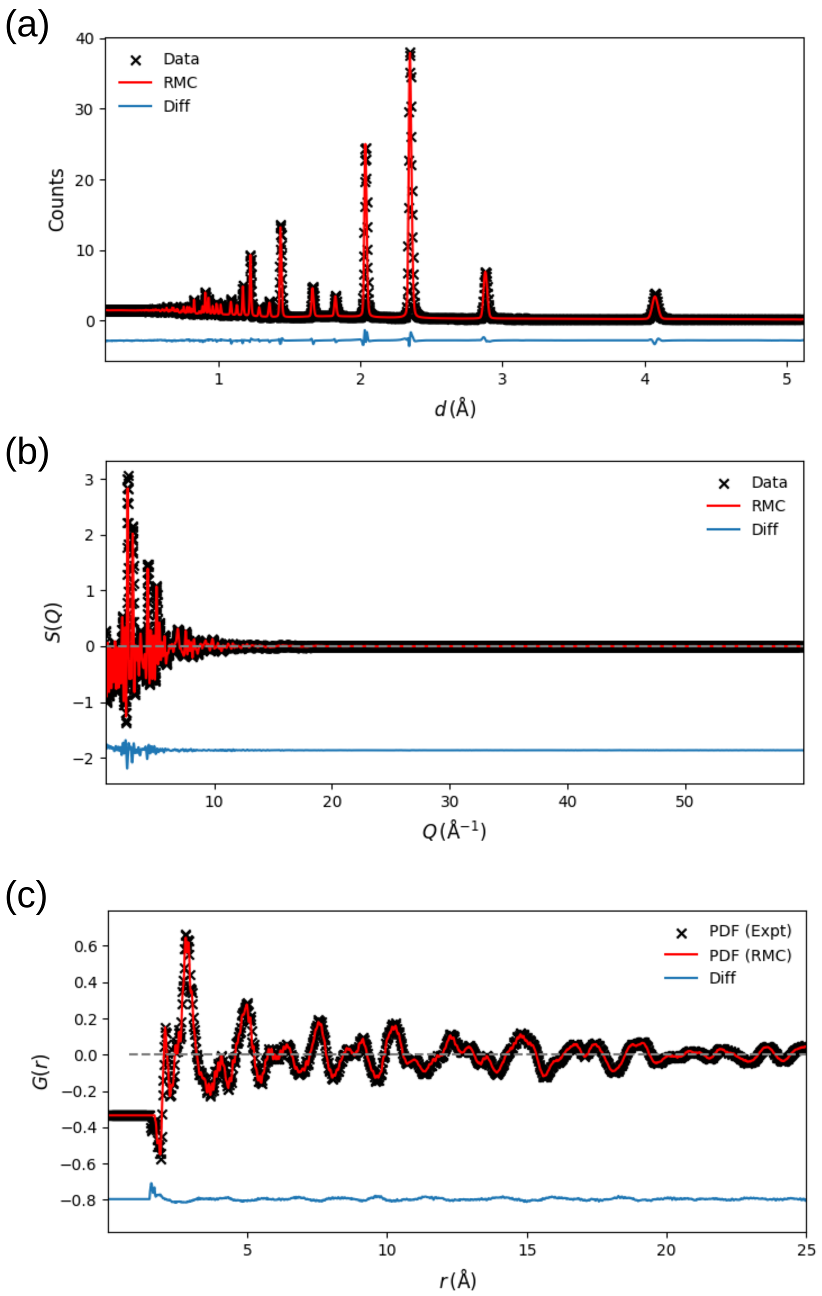

Fig. S8 Fitted (a) Bragg (b) $S(Q)$ and (c) $G(r)$ data for poled 5PMS-PZT using the RMC method.

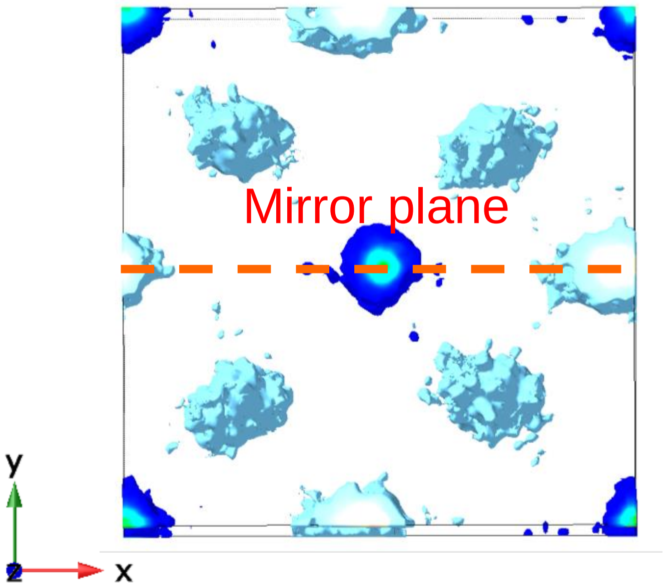

Fig. S9 Oxygen atom density in folded-back supercell illustrating preservation of the mirror plane perpendicular to the monoclinic *b*-axis.

(a)

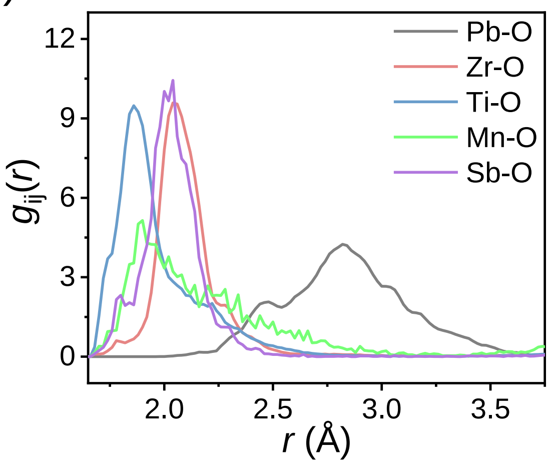

(b)

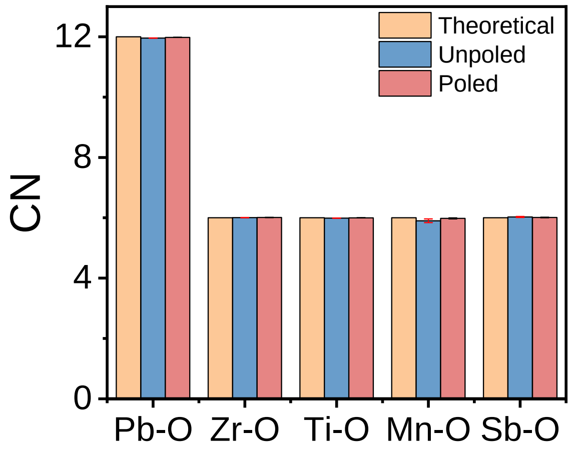

Fig. S10 (a) Partial correlation functions (1.5 Å < r < 3.75 Å) for M-O pairs for poled 5PMS-PZT, (b) Comparison of the first-nearest coordination number (CN) of metal atoms against theoretical values, indicating negligible oxygen deficiency.

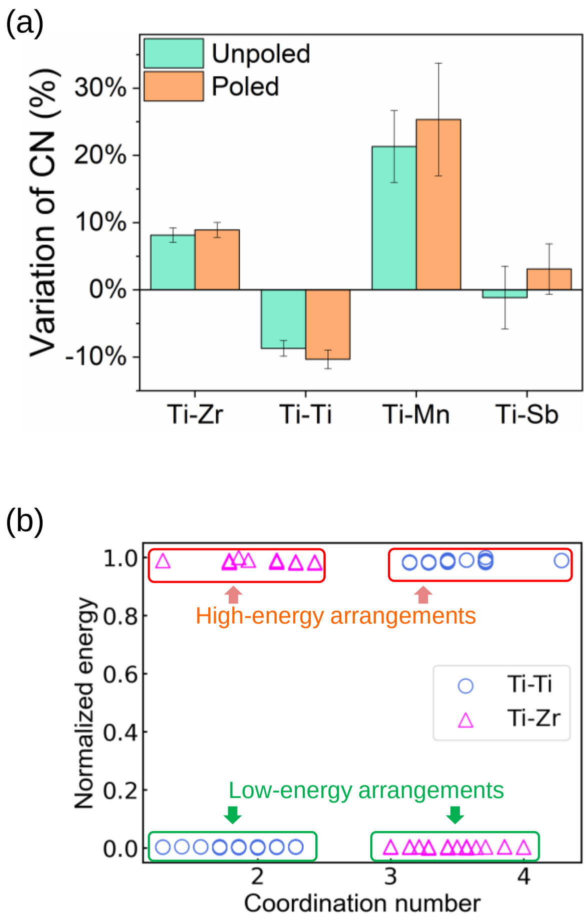

Fig. S11 (a) Variation of coordination number (CN) in percentages for Ti in the 3rd-nearest coordination shell. (b) Calculated electrostatic energy as a function of Ti CN, demonstrating energetic favorability of Zr-rich environments.

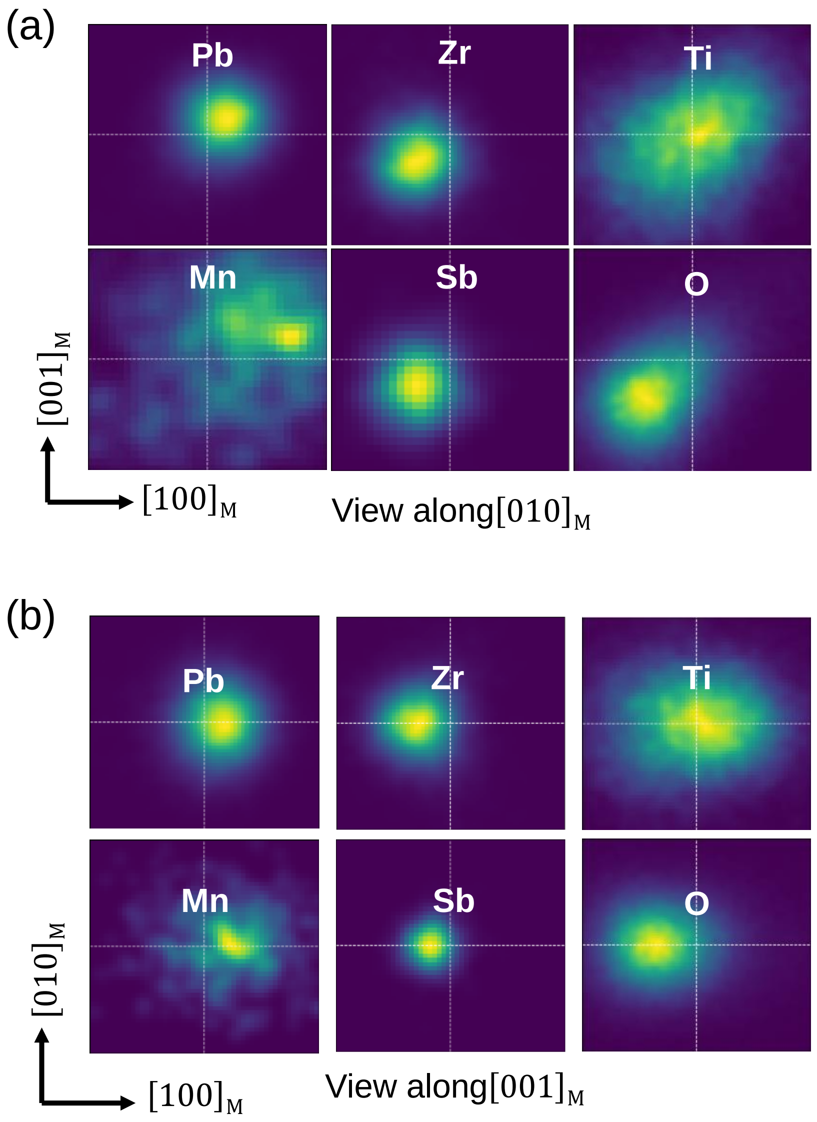

Fig. 12 Projected densities of atomic off-center displacements in poled structure onto the (a) *a*-*c* plane and (b) *a*-*b* plane.

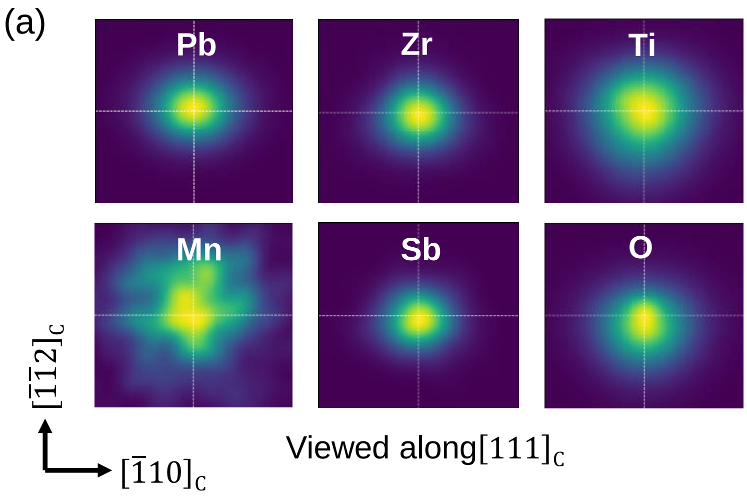

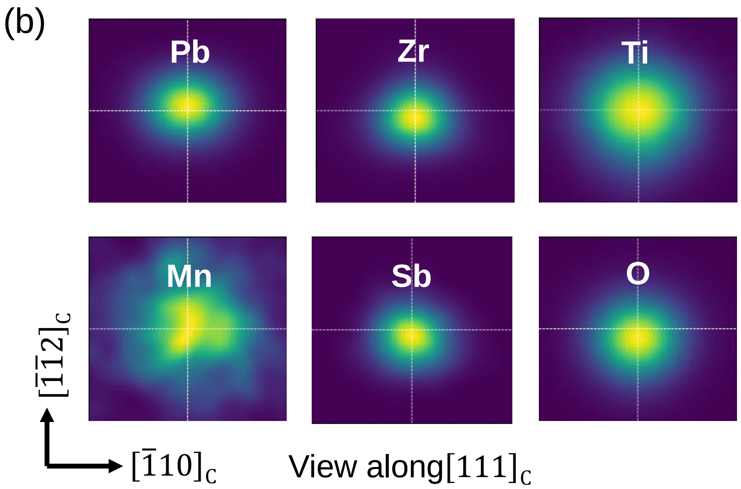

Fig. S13 Projected densities of atomic off-center displacements in (a) unpoled and (b) poled structures viewed along the $[111]_C$ direction.

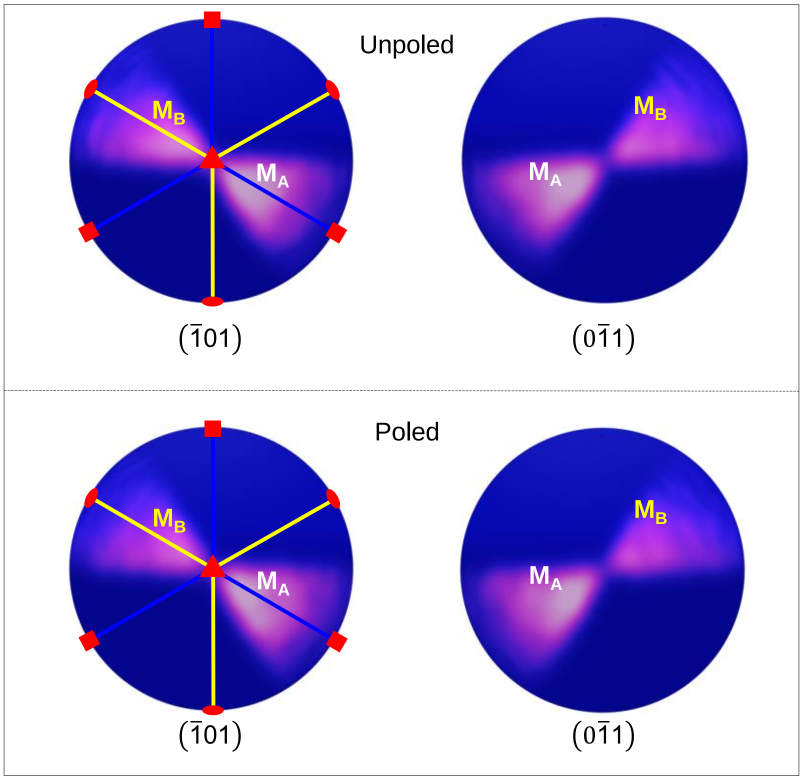

Fig. S14 Spherical projections of local polarization vectors associated with $(\bar{1}01)$ and $(0\bar{1}1)$ reflections in unpoled and poled structures. Vectors are found in both M_A and M_B regions, while the M_A region show more intensive densities.

Tables

Table S1. Crystal and refinement parameters for unpoled and poled 5PMS-PZT at room temperature based on fits to neutron diffraction data.

Chemical formula	$PbZr_{0.494}Ti_{0.456}Mn_{0.0167}Sb_{0.0333}O_6$	
Poling state	Unpoled	Poled
Space group	*Cm*	*Cm*
Lattice parameters (Å)	a = 5.7556(4)<br>b = 5.7375(4)<br>c = 4.0842(3)<br>β = 90.402(5)°	a = 5.7535(4)<br>b = 5.7355(4)<br>c = 4.0770(4)<br>β = 90.437(5)°
Volume (Å^3)	134.87(2)	134.53(2)
Z	2	2
Density (calc) g cm^{-3}	8.058	8.074
R-factors	R_{wp} = 0.0440<br>R_p = 0.0332<br>R_{ex} = 0.0026<br>R_{F^2} = 0.208	R_{wp} = 0.0400<br>R_p = 0.0303<br>R_{ex} = 0.0024<br>R_{F^2} = 0.2030
No. of variables	56	56
No. of profile points	3212	3212

Table S2. Refined atomic parameters for unpoled and poled 5PMS-PZT using *Cm* model.

(a)

	Atom	Wyckoff	*x*	*y*	*z*	Occ.	U_{iso}
Unpoled	Pb	2*a*	0	0	0	1.0	0.0261(7)
	Zr	2*a*	0.467(3)	0.0	0.551(4)	0.494	0.02
	Ti	2*a*	0.467(3)	0.0	0.551(4)	0.456	0.02
	Mn	2*a*	0.467(3)	0.0	0.551(4)	0.017	0.02
	Sb	2*a*	0.467(3)	0.0	0.551(4)	0.033	0.02
	O1	2*a*	0.453(1)	0.0	0.064(2)	1.0	0.040(2)
	O2	4*b*	0. 2061(9)	0.2435(4)	0.582(1)	1.0	0.0120(5)

(b)

	Atom	Wyckoff	*x*	*y*	*z*	Occ.	U_{iso}
Poled	Pb	2*a*	0	0	0	1.0	0.0308(7)
	Zr	2*a*	0.464(2)	0.0	0.547(4)	0.494	0.01
	Ti	2*a*	0.464(2)	0.0	0.547(4)	0.456	0.01
	Mn	2*a*	0.464(2)	0.0	0.547(4)	0.017	0.01
	Sb	2*a*	0.464(2)	0.0	0.547(4)	0.033	0.01
	O1	2*a*	0.4493(9)	0.0	0.062(2)	1.0	0.036(1)
	O2	4*b*	0.2027(8)	0.2438(3)	0.579(1)	1.0	0.0129(4)

Table S3. Selected bond lengths and angles in the monoclinic phase of unpoled and poled 5PMS-PZT.

(a)	Bond length (Å)				
Bonds	Pb-O1	Pb-O1(×2)	Pb-O2(×2)	Pb-O2(×2)	Pb-O2(×2)
Unpoled	2.608(7)	2.8938(8)	2.512(3)	2.986(5)	2.819(5)
Poled	2.596(5)	2.8940(9)	2.509(4)	2.973(5)	2.827(5)
Bonds	B_{site}-O1	B_{site}-O1′	2 × B_{site}-O2	2 × B_{site}-O2′	
Unpoled	2.00(2)	2.09(2)	2.06(1)	2.01(1)	
Poled	1.98(2)	2.10(1)	2.058(9)	2.01(1)	

(b)	Bond angle (º)			
Angles	O1-B_{site}-O2 (×2)	O1-B_{site}-O2 (×2)	O1-B_{site}-O2 (×2)	O1-B_{site}-O2 (×2)
Unpoled	91.4(9)	94.8(2)	84.6(2)	88.8(8)
Poled	92.1(8)	95.0(1)	84.4(2)	88.2(7)
Angles	O2-B_{site}-O2	O2-B_{site}-O2 (×2)	O2-B_{site}-O2	
Unpoled	85.2(6)	89.95(2)	94.2(7)	
Poled	85.6(5)	89.91(3)	93.7(6)	

Note: (×2) means there are two angles with the same value.

Table S4. Atomic displacements (Å) in the equivalent pseudo-cubic unit cell for unpoled and poled 5PMS-PZT.

	Atom	dx	dy	dz	|dx|/|dz|
Unpoled	Pb	0	0	0	~
	B-site	-0.190	-0.190	0.208	0.912
	O	-0.259	-0.259	0.310	0.833
Poled	Pb	0	0	0	~
	B-site	-0.207	-0.207	0.192	1.081
	O	-0.279	-0.279	0.299	0.932

Note: in refinements Pb was fixed at the origin of the cell.

Table S5. Neutron coherent scattering length (bcoh) and cross section (σcoh) values for elements in 5PMS-PZT.

	Element	b_{coh}	σ_{coh}	Occupancy
A-site	Pb	9.405	11.115	1
B-site	Zr	7.16	6.44	0.494
	Ti	-3.438	1.485	0.456
	Mn	-3.73	1.75	0.017
	Sb	5.57	3.9	0.033
	Average	2.089	4.017	1
O		5.803	4.232	1

Note: b_{coh} and σ_{coh} were obtained from https://www.ncnr.nist.gov/resources/n-lengths.

Table S6. Peak and average contact distances for Pb-O and B-O pairs obtained from RMC and DFT calculations v.s. the average distances obtained from refinements.

Sample	Methods	Distance values for atom pairs				
Unpoled		Pb-O	Zr-O	Ti-O	Mn-O	Sb-O
	Theoretical	2.84	2.07	1.955	2.02	1.95
	RMC model	2.87(2)	2.125(2)	2.011(2)	2.201(7)	2.051(3)
			Mean (B-O): 2.072			
	Crystallographic model	2.89(26)	2.04(4)			
	DFT model	2.84	2.08	1.98	1.99	1.99
			Mean (B-O): 2.03			
Poled		Pb-O	Zr-O	Ti-O	Mn-O	Sb-O
	RMC model	2.801(5)	2.096(3)	1.852(3)	1.83(4)	2.00(2)
		2.901(2)	2.127(1)	2.02(4)	2.187(9)	2.059(3)
			Mean(B-O): 2.077			
	Crystallographic model	2.89(28)	2.04(4)			

Note: theoretical bond lengths[16] were calculated by using the ionic radii of each type of ion in a certain coordination environment, namely, $r(Pb^{2+})$ = 1.49 Å with coordination number (CN) of 12, $r(O^{2-})$ = 1.35 Å with CN of 2, and all B-site cations $r(Zr^{4+})$ = 0.72 Å, $r(Ti^{4+})$ = 0.605 Å, $r(Mn^{2+})$ = 0.67 Å and $r(Sb^{5+})$ = 0.60 Å with CN of 6.

Table S7. Averaged angular distortion indices (D_A) and bond distortion indices (D_B) for BO_6 octahedra in RMC models for unpoled and poled 5PMS-PZ. The indices obtained from DFT simulation are also shown but only in the unpoled state.

		Octahedra	ZrO_6	TiO_6	MnO_6	SbO_6
Unpoled	RMC	D_A	0.086(2)	0.104(2)	0.169(8)	0.074(4)
		Contributions	44.7%	49.7%	3.0%	2.6%
		Overall D_A	0.095(2)			
	DFT	D_A	0.056	0.061	0.016	0.029
		Contributions	47.3%	49.5%	0.5%	2.7%
		Overall D_A	0.057			
Poled	RMC	D_A	0.0933(9)	0.122(3)	0.184(7)	0.077(3)
		Contributions	45.0%	49.6%	3.0%	2.5%
		Overall D_A	0.103(1)			
Unpoled	RMC	D_B	0.0482(7)	0.084(1)	0.104 (4)	0.045(2)
		Contributions	34.3%	55.4%	2.5%	2.1%
		Overall D_B	0.0654(9)			
	DFT	D_B	0.029	0.058	0.012	0.014
		Contributions	33.2%	63.9%	0.5%	2.4%
		Overall D_B	0.042			
Poled	RMC	D_B	0.0545(6)	0.0894(7)	0.119(6)	0.048(2)
		Contributions	37.8%	57.2%	2.8%	2.2%
		Overall D_B	0.0713(8)			

Table S8. Simulated charges using DFT showing covalency of atoms in the RMC model.

Element	Pb	Zr	Ti	Mn	Sb	O
Charge	1.36(2)	2.65(2)	2.35(2)	1.54(7)	2.73(6)	-1.28(3)

Equation S1:

$$\begin{bmatrix} a_M \\ b_M \\ c_M \end{bmatrix} = \begin{bmatrix} 1 & 1 & 0 \\ -1 & 1 & 0 \\ 0 & 0 & 1 \end{bmatrix} \begin{bmatrix} a_c \\ b_c \\ c_c \end{bmatrix} \qquad (1)$$

where $\boldsymbol{a}_M$, $\boldsymbol{b}_M$ and $\boldsymbol{c}_M$ represent the basis vectors for the monoclinic unit cell, and $\boldsymbol{a}_c$, $\boldsymbol{b}_c$ and $\boldsymbol{c}_c$ for the pseudo-cubic cell. With this relationship, it is easy to convert a vector or coordinates between the two models. For example, a vector in the monoclinic cell, $\vec{u}_M = [m,n,p]$ can be expressed with respect to the cubic cell by $\vec{u}_M = m\vec{a}_M + n\vec{b}_M + p\vec{c}_M = (m-n)\vec{a}_c + (m+n)\vec{b}_c + p\vec{c}_c$ and vice versa, e.g. $\vec{v}_c = [m,n,p]$ in the cubic cell can be expressed by $\vec{v}_c = m\vec{a}_c + n\vec{b}_c + p\vec{c}_c = 0.5(m-n)\vec{a}_M + 0.5(m+n)\vec{b}_M + p\vec{c}_M$

For convenience, the equivalent expressions for crystallographic directions in the two unit cells are listed in the following table:

Monoclinic	100	010	001	110	101	011	111	$\bar{1}10$	$\bar{1}01$	$\bar{3}12$	201
Cubic	110	$\bar{1}10$	001	020	111	$\bar{1}10$	021	200	$\bar{1}11$	211	221

Equation S2:

With known atomic positions in a unit cell, the theoretical spontaneous polarizations (P_s) can be calculated through the following equation[17]:

$$P_s = \sum_i \frac{n_i \Delta x_i Q_i e}{V} \qquad (2)$$

where n_i is the number of atom type i in the unit cell, Δx_i off-center displacement, $Q_i e$ the ionic charge and V volume of the cell.

Equation S3:

The *CN* of atom type *i* with coordination to atom type *j* in the distance range from r_1 to r_2 can be calculated by integrating the partial radial distribution function, $g_{ij}(r)$[18], as follows:

$$CN = \int_{r_1}^{r_2} 4\pi r^2 \rho_0 c_j g_{ij}(r) dr \quad (3)$$

where *r* is the searching distance from the center atom, c_j is the proportion of atom type *j* in the material, ρ_0 is atomic number density and $g_{ij}(r)$ is the partial correlation function. Usually, the first peak in $g_{ij}(r)$ is considered in the calculation of *CN*, because it represents the first nearest coordination shell of the center atom.

Equation S4:

In the perovskite structure, one B-site cation is surrounded by 6 other B-site cations. It is easy to calculate the theoretical coordination number for a certain type of B-site cation based on the concentration of neighbouring cations through the formula:

$$CN_{(A-B)} = 6 \times C_B \tag{4}$$

where $CN_{(A\text{-}B)}$ is the number of cation B around cation A and C_B is the concentration of cation B. The theoretical *CN*s for any B-site cation with coordination to Zr, Ti, Mn and Sb are therefore 2.964, 2.736, 0.1 and 0.2.

Equations S5 and S6:

The distortion of an octahedron in the ABO_3 perovskite structure can be evaluated by two indices[19], namely the angular distortion index D_A, and the bond length distortion index D_B, with the expressions as:

$$D_A = \frac{\sum_{ij} |\angle O_iBO_j - \theta_m|}{12\theta_m} \tag{S5}$$

and

$$D_B = \frac{\sum_i |l_i - l_m|}{6 l_m} \tag{S6}$$

where $\angle O_iBO_j$ is the angle between B-O_i and B-O_j bonds, and m represents the mean angle. The summation of D_A is taken over all neighboring O atoms i and j, involving 12 independent bond angles in the octahedron. The term l_i represents the bond length between B-site atom and the ith oxygen O_i, while l_m is the mean bond length.[19]

Equation S7:

Percent ionic character (%IC) of a bond between atoms A and B can be approximated by the equation:

$$\%IC = [1 - e^{\text{[illegible]}}] \times 100 \qquad (7)$$

where X_A and X_B are the electronegativities of atoms A and B.

Equation S8:

The displacive site disorder of atoms can be evaluated by the root mean square displacement factor using:

$$RMSD = \sqrt{\frac{1}{N}\sum_{i=1}^{N}\left(\Delta x_i^2 + \Delta y_i^2 + \Delta z_i^2\right)} \qquad (S8)$$

where Δx_i, Δy_i, and Δz_i are the displacements of atom i from its ideal position. N is the total number of atoms. A higher RMSD can be induced by stronger local off-centering of atoms.

Equation S9:

The relationships for different formalisms of correlation functions were summarized in D. Keen's paper.[18] The correlation functions used in this work are $G^{PDF}(r)$ and $G(r)$, which have the inner relationship with the radial distribution function $D(r)$ as below:

$$G^{PDF}(r) = \frac{4\pi\rho_0 r G(r)}{\left(\sum_i c_i \bar{b}_i\right)^2} = \frac{D(r)}{\left(\sum_i c_i \bar{b}_i\right)^2} \tag{9}$$

where c_i and $\bar{b}_i$ are the molar concentration and neutron coherent scattering length for atom species i, respectively, and ρ_0 is number density of the unit cell.

References

1. De Backer, A.; Van den Bos, K.; Van den Broek, W.; Sijbers, J.; Van Aert, S., StatSTEM: An efficient approach for accurate and precise model-based quantification of atomic resolution electron microscopy images. *Ultramicroscopy* **2016,** *171*, 104-116.
2. Van Aert, S.; De Backer, A.; Martinez, G. T.; Den Dekker, A. J.; Van Dyck, D.; Bals, S.; Van Tendeloo, G., Advanced electron crystallography through model-based imaging. *IUCrJ* **2016,** *3* (1), 71-83.
3. Fu, Z.; Chen, X.; Nie, H.; Liu, Y.; Hong, J.; Hu, T.; Yu, Z.; Li, Z.; Zhang, L.; Yao, H., Atomic reconfiguration among tri-state transition at ferroelectric/antiferroelectric phase boundaries in Pb(Zr,Ti)O3. *Nat. Commun.* **2022,** *13* (1), 1390.
4. Larson, A. C.; Von Dreele, R. B. *GSAS Generalised Structure Analysis System*; Los Alamos National Laboratory Report LAUR-86-748; Los Alamos National Laboratory, 1987.
5. Toby, B. H., EXPGUI, a graphical user interface for GSAS. *Appl. Crystallogr.* **2001,** *34* (2), 210-213.
6. Frantti, J.; Lappalainen, J.; Eriksson, S.; Lantto, V.; Nishio, S.; Kakihana, M.; Ivanov, S.; Rundlöf, H., Neutron diffraction studies of Pb(ZrxTi1-x)O3 ceramics. *Jpn. J. Appl. Phys.* **2000,** *39* (9B), 5697.
7. Michel, C.; Moreau, J. M.; Achenbach, G. D.; Gerson, R.; James, W. J., Atomic structures of two rhombohedral ferroelectric phases in the Pb(Zr, Ti)O3 solid solution series. *Solid State Commun.* **1969,** *7* (12), 865-868.
8. Buttner, R.; Maslen, E., Structural parameters and electron difference density in BaTiO3. *Structural Science* **1992,** *48* (6), 764-769.
9. Tucker, M. G.; Keen, D. A.; Dove, M. T.; Goodwin, A. L.; Hui, Q., RMCProfile: reverse Monte Carlo for polycrystalline materials. *J. Phys.: Condens. Matter* **2007,** *19* (33), 335218.
10. Farrow, C.; Juhas, P.; Liu, J.; Bryndin, D.; Božin, E.; Bloch, J.; Proffen, T.; Billinge, S., PDFfit2 and PDFgui: computer programs for studying nanostructure in crystals. *J. Phys.: Condens. Matter* **2007,** *19* (33), 335219.
11. Okhotnikov, K.; Charpentier, T.; Cadars, S., Supercell program: a combinatorial structure-generation approach for the local-level modeling of atomic substitutions and partial occupancies in crystals. *Journal of cheminformatics* **2016,** *8*, 1-15.
12. Kresse, G.; Hafner, J., Ab initio molecular dynamics for liquid metals. *Phys. Rev. B,* **1993,** *47* (1), 558.
13. Kresse, G.; Joubert, D., From ultrasoft pseudopotentials to the projector augmented-wave method. *Phys. Rev. B,* **1999,** *59* (3), 1758.
14. Furness, J. W.; Kaplan, A. D.; Ning, J.; Perdew, J. P.; Sun, J., Accurate and numerically efficient r2SCAN meta-generalized gradient approximation. *The journal of physical chemistry letters* **2020,** *11* (19), 8208-8215.
15. Dudarev, S. L.; Botton, G. A.; Savrasov, S. Y.; Humphreys, C.; Sutton, A. P., Electron-energy-loss spectra and the structural stability of nickel oxide: An LSDA+ U study. *Phys. Rev. B,* **1998,** *57* (3), 1505.
16. Shannon, R. D., Revised effective ionic radii and systematic studies of interatomie distances in halides and chaleogenides. *Acta Cryst.* **1976,** *A* (32), 751-767.

17. Zhang, H.; Krynski, M.; Fortes, A. D.; Saunders, T. G.; Palma, M.; Hao, Y.; Krok, F.; Yan, H.; Abrahams, I., Origin of polarization in bismuth sodium titanate-based ceramics. *J. Am. Chem. Soc.* **2024,** *146* (8), 5569-5579.
18. Keen, D. A., A comparison of various commonly used correlation functions for describing total scattering. *Appl. Crystallogr.* **2001,** *34* (2), 172-177.
19. Baur, W., The geometry of polyhedral distortions. Predictive relationships for the phosphate group. *Structural Science* **1974,** *30* (5), 1195-1215.
